# Small extracellular vesicle-mediated miR-320e transmission promotes osteogenesis in OPLL by targeting TAK1

**DOI:** 10.1038/s41467-022-29029-6

**Published:** 2022-05-05

**Authors:** Chen Xu, Zicheng Zhang, Ning Liu, Li Li, Huajian Zhong, Ruizhe Wang, Qianghui Shi, Zifan Zhang, Leixin Wei, Bo Hu, Hao Zhang, Xiaolong Shen, Yue Wang, Yang Liu, Wen Yuan

**Affiliations:** 1 Department of Orthopedics, Shanghai Changzheng Hospital, Naval Medical University, Shanghai, 200003 China; 2 Department of Orthopedics, the Fouth Medical Center of PLA General Hospital, Beijing, 100048 China; 3 Department of Histology and Embryology, College of Basic Medicine, Naval Medical University, Shanghai, 200433 China; 4 Department of Orthopedics, 923th Hospital of the Joint Logistics Support Force of PLA, Nanning, 530021 China; 5 Department of Orthopedics, 967th Hospital of the Joint Logistics Support Force of PLA, Dalian, 116021 China

**Keywords:** miRNAs, Bone, Golgi

## Abstract

Ossification of the posterior longitudinal ligament (OPLL) is an emerging spinal disease caused by heterotopic ossification of the posterior longitudinal ligament. The pathological mechanism is poorly understood, which hinders the development of nonsurgical treatments. Here, we set out to explore the function and mechanism of small extracellular vesicles (sEVs) in OPLL. Global miRNA sequencings are performed on sEVs derived from ligament cells of normal and OPLL patients, and we have showed that miR-320e is abundantly expressed in OPLL-derived sEVs compare to other sEVs. Treatment with either sEVs or miR-320e significantly promote the osteoblastic differentiation of normal longitudinal ligament cells and mesenchymal stem cells and inhibit the osteoclastic differentiation of monocytes. Through a mechanistic study, we find that TAK1 is a downstream target of miR-320e, and we further validate these findings in vivo using OPLL model mice. Together, our data demonstrate that OPLL ligament cells secrete ossification-promoting sEVs that contribute to the development of ossification through the miR-320e/TAK1 axis.

## Abstract (web summary)

The pathological mechanisms that lead to Ossification of the posterior longitudinal ligament (OPLL) are unclear. Here, the authors show that OPLL ligament cells produce small extracellular vesicles that induce ossification via miR-320e/TAK1 signaling in mice and human posterior longitudinal ligament cells.

## Introduction

Ossification of the posterior longitudinal ligament (OPLL) is a common spinal disease involving ectopic ossification of the posterior longitudinal ligament, and it is often accompanied by severe compression of the spinal cord and nerve roots, resulting in quadriparesis or other myelopathy manifestations^1,2^. In the early stages of the disease, the ossification of the ligament advances slowly, and most patients are not aware of the disease until they develop obvious myelopathy symptoms due to the large osteophytes that progress over time. Thus, patients with late-stage OPLL are often hospitalized, and the clinical outcome of these patients is usually not satisfactory^3^. Moreover, the molecular pathogenesis has not been elucidated and no efficient therapeutic strategy, especially pharmacotherapy and/or a preventive intervention for OPLL, has been suggested, making surgical management by indirect decompression of the spinal cord the only option for symptomatic OPLL patients.

OPLL is known to be an idiopathic and multifactorial disease in which familial inheritance (genetic factors) and nongenetic factors, including diet, obesity, physical strain on the posterior longitudinal ligament, age, and diabetes mellitus, are involved in the pathogenesis^4^. However, the detailed mechanism remains unclear. Studies have shown that various single nucleotide polymorphisms (SNPs) of osteogenesis-related genes are related to the development of abnormal ossification in OPLL^5–9^; however, they were also found to play critical roles in normal ossification processes, such as endochondral bone formation or osteocyte differentiation (such as *Col6a1, Tgf-β1, Npp1, Bmp2*, etc.)^10–14^. This phenomenon implies that key disease-specific factors or regulatory networks are still being unraveled in the development of OPLL. A recent genome-wide association study identified new disease-related loci^15^ and new OPLL-specific factors related to disease development^16^.

Studies have shown that aside from potential genetic variations, mechanical stress, dietary factors and focal inflammatory responses also contribute to the development of OPLL. These environmental factors indicate that an imbalance in homeostasis of the ligament environment may also be important in the pathogenesis of OPLL^17^. Recent findings have shown that extracellular vesicles (EVs) are vital functional players that contribute to the pathogenesis of many diseases^18^. EVs are membrane-containing vesicles released in an evolutionarily conserved manner by cells, ranging from organisms such as prokaryotes to higher eukaryotes and plants^19^. Small EVs (sEVs) ranging from 50 nm–200 nm in size have been widely studied and are considered a vital medium for intercellular signal transduction. These signals can be transmitted by sEVs via different biomolecules, such as proteins, lipids, nucleic acids and sugars. The unique package containing this information provides both protection and the option of simultaneous delivery of multiple different messengers, even to sites remote from its origin. Small RNAs such as microRNAs (miRNAs) are the most commonly studied sEV content and play vital roles in various biological or pathological processes, such as tumor metastasis^20^, bone formation^21^, and neurological diseases^22,23^. Since reports have shown that sEVs may contribute to the bone formation process by regulating extracellular mineral formation, we hypothesized that sEVs may also contribute to heterotopic ossification processes such as OPLL development, which has not yet been clearly revealed.

In our previous study, we found that OPLL cellular miRNAs might be upstream mediators that play important roles in the ossification process of ligament cells. However, how these miRNAs contribute to disease development has not been elucidated. Here, we report that small EVs derived from OPLL significantly promote the osteogenic differentiation of posterior longitudinal ligament (PLL) cells and mesenchymal stem cells (MSCs) and inhibit monocyte osteoclast differentiation both in vitro and in vivo. By using high-throughput miRNA sequencing data and bioinformatics target prediction and validation, we found that miR-320e-induced transforming growth factor-beta-activated kinase-1 (TAK1) was responsible for this effect, and miR-320e was uniquely aggregated in OPLL-derived sEVs compared to its cellular hosts. Our findings demonstrate that disease-specific miRNAs could be delivered to the nearby cells by OPLL cells via small EV secretion, thus elevating the osteogenic potentials of normal ligament cells and MSCs to contribute to the development of OPLL. Our results also highlight that OPLL-secreted sEVs inhibit the osteoclastogenesis of monocytes, which further alters the homeostasis of the ligament towards an OPLL-specific pathogenic microenvironment.

## Results

### Isolation and identification of OPLL derived small extracellular vesicles

**Fig. 1:**
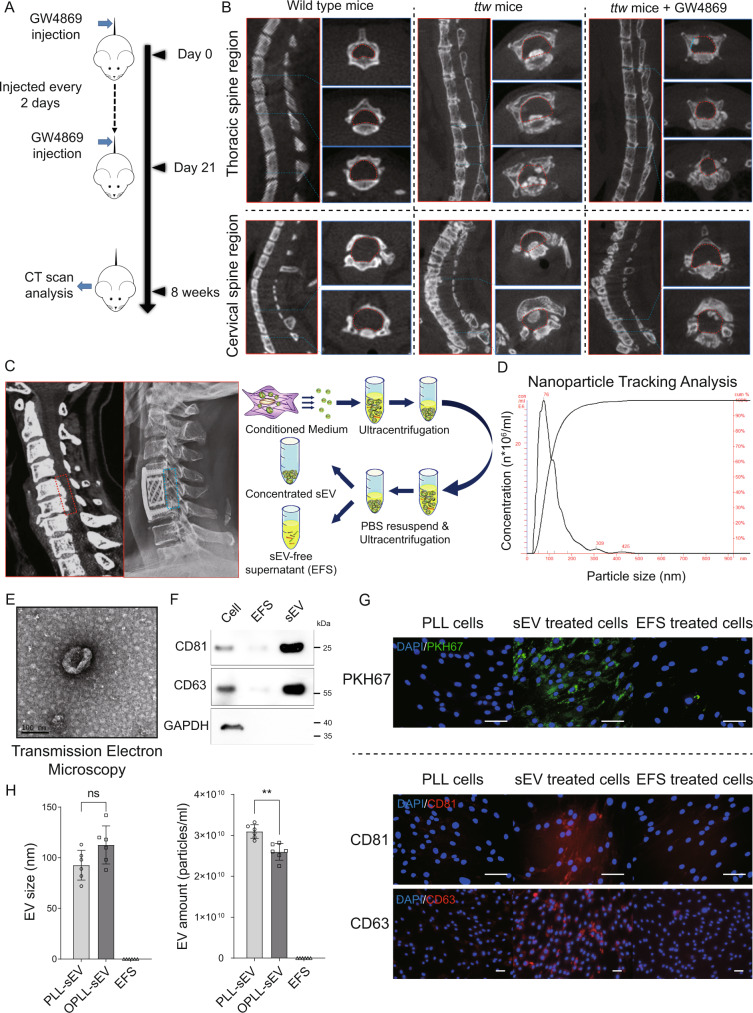
Small extracellular vesicles (sEVs) are vital to the development of OPLL. **A** Scheme of GW4869 injection in *ttw* mice to examine the effect of sEVs inhibition on OPLL development. **B** Computed tomography showing the occupation of ossified ligament tissue in the spinal canal of *ttw* mice. The red dashed line indicates the area of spinal canal. **C** Typical CT image of cervical OPLL patient (left panel with red dashed square indicate OPLL in the spine canal), and postoperative X-ray image showing the resection of the vertebrate and the ossified ligament tissue (left panel with blue dashed square). The workflow showing how sEVs are collected and purified from OPLL and normal PLL ligament cell supernatant was shown in the right panel. **D** The Nanoparticle Tracking Analysis (NTA) showing the particle size/concentration (left panel) and particle size/relative intensity (right panel) plot of the collected sEVs. Note that most detected particles were around 100 nm in size. **E** The Transmission Electron Microscopy (TEM) analysis showing the typical image of sEV collected in the experiment was shown. The scale bar represents 100 nm, *n* = 6 biologically independent repeats with similar results. **F** Western blot analysis confirmed the extracellular vesicle marker expression in ligament cells (Cell group), EV-free supernatant (EFS group) and sEV group, *n* = 3 biologically independent repeats with similar results. **G** sEV uptake analysis was performed and analyzed using immunofluorescence microscopy. sEVs were labeled with membrane-specific dye PKH67 and purified before incubation with ligament cells for 6 h. Cells were washed, fixed and counterstained with DAPI (upper panel). **G** Non-labeled sEV and EFS were incubated with ligament cells for 6 h, and after wash, the cells were fixed and immunocytochemistry analysis was performed to show cellular expression of CD81 and CD63 using specific antibodies (lower panel). EFS represented the collected supernatant that free of sEVs. The scale bar represents 100 μm. **H** Comparison of the sEV size and amount data between PLL and OPLL ligament cell derived sEVs recorded from NTA analysis. EFS represented the collected supernatant that free of sEVs. Data were presented as mean ± SD, *n* = 6 biologically independent samples, **p = 0.01, *t* test two tailed; ns not significant, *p* = 0.0692. Source data are provided as a Source Data file.

Small extracellular vesicles (sEVs) range from 50–200 nm in size. To explore the function of OPLL-derived small EVs, we first performed sEV secretion inhibition by administrating GW4869 into *ttw* mice (OPLL model mice) through tail veins (Fig. 1A). After 8 weeks, the micro-CT scans were performed and we found that GW4869 administration significantly reduced heterotopic ossification of the posterior longitudinal ligament compared to control *ttw* mice, while wild type ICR mice showed no ossification (Fig. 1B, S1A). To further explore the function of OPLL ligament cell derived sEV, we performed ultracentrifugation to collect small EVs from primary OPLL cells isolated from OPLL patients received anterior corpectomy and fusion surgery as previously described^24,25^ (Fig. 1C). The isolated sEVs were first identified by nanoparticle tracking analysis (NTA) to determine their size. The results showed that the particles collected were ~100 nm in size (Fig. 1D, S1B), and transmission electron microscopy (TEM) analysis showed particles with typical sEV morphology (Fig. 1E). Further western blot analysis confirmed that the collected sEVs expressed abundant CD81 and CD63 (Fig. 1F), common markers used for sEV identification. To identify whether the collected EVs are able to transmit to other cells, we performed PKH67 uptake analysis on PLL ligament cells, and we could observe strong PKH67 dye around the cytoplasm of EV treated PLL cells (Fig. 1G, upper panel). Further immunofluorescence analysis identified increased CD81 and CD63 expression in EV treated PLL cells (Fig. 1G, lower panel). To gain further information between OPLL derived and PLL derived small EVs, we compared the EV size and amount from OPLL and PLL groups, and found that although they showed no significant differences in size, the amount of EVs secreted by PLL ligament cells were significantly more than that of OPLL ligament cells (Fig. 1). This may be related to the different proliferation rates between PLL and OPLL cells^26^. Collectively, we showed that small EV secretion is important for OPLL development, and we have performed initial identification of OPLL and PLL ligament cell derived small EVs which will be used for further analysis.

### The global micronome revealed OPLL-sEV specific microRNAs

**Fig. 2:**
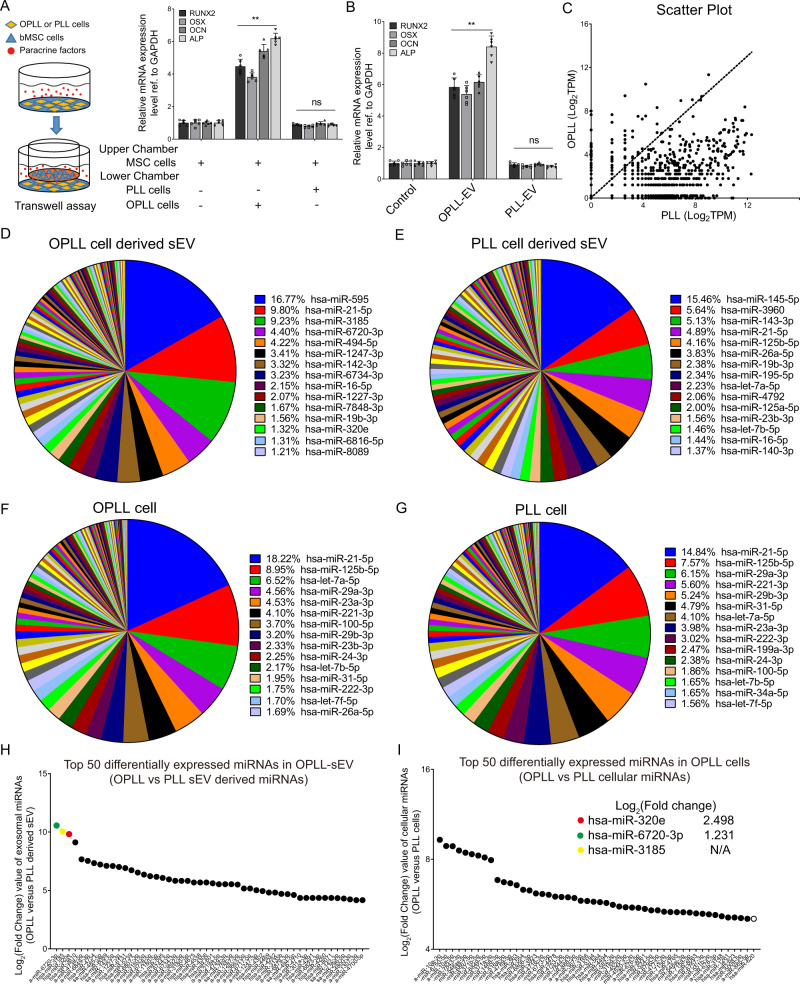
Micronome analysis identified OPLL-sEV specific microRNAs. **A** Transwell assay followed by qRT-PCR detecting the expression changes of osteogenic genes in Mesenchymal stem cells (MSCs) co-cultured with or without PLL and OPLL cells. The scheme was shown in the left panel, *n* = 6 biologically independent samples, gene expression in each group was compared to MSC alone group using two tailed *t* test, **p = 0.0001; ns not significant, *p* = 0.999. **B** qRT-PCR analysis detecting the expression changes of osteogenic genes in MSCs treated with OPLL (OPLL-sEV) or PLL (PLL-sEV) derived sEVs, *n* = 6 biologically independent samples, gene expression in each group was compared to control group using two tailed *t* test, **p = 0.0001; ns not significant, *p* = 0.999. High through-put microRNA (miRNAs) sequencing was performed in OPLL and PLL derived sEVs, and the scatter plot displayed the differentially expressed miRNAs between OPLL and PLL derived sEVs (**C**). The abundance of expressed miRNAs is shown in percentage in the pie chart of OPLL derived sEVs (**D**) and PLL derived sEVs (**E**), while data of OPLL (**F**) and PLL cells (**G**) were also displayed. Note that significant differences were found in top 15 abundant miRNAs between OPLL or PLL cell and derived sEVs. The top 50 differentially expressed miRNAs were shown between OPLL derived sEVs and PLL derived sEVs (**H**) or OPLL cells and PLL cells (**I**). The expression fold change of the top three differentially expressed miRNAs were listed in (**I**), as they are not found in the cellular level of top 50 differentially expressed miRNAs. All qPCR data were presented as mean ± SD, and GAPDH level were detected and served as internal reference. Source data are provided as a Source Data file.

sEVs are known to be important regulators of intercellular communication^27^. We further performed Transwell system-facilitated coculture assay to determine whether OPLL cell-secreted factors may influence the ossification process (Fig. 2A). Using qRT-PCR analysis, we found that ossification-related genes were significantly upregulated only when OPLL cells were cocultured with mesenchymal stem cells (MSCs) (Fig. 2A). By directly adding OPLL or PLL ligament cell-derived sEVs into MSCs, we also found that OPLL-sEVs significantly increased the expression of ossification-related genes (Fig. 2B). These results provide initial insight into the function of OPLL-derived sEVs. To explore in depth, we performed high-throughput small RNA sequencing and examined the global microRNA profile of OPLL- and PLL-derived sEVs (Fig. S2A). The scatter plot revealed significant differences in the microRNA expression profiles between OPLL- and PLL-derived sEVs (Fig. 2C). By comparing the abundantly expressed microRNAs in OPLL- and PLL-derived sEVs, we found that both OPLL- and PLL-derived sEVs had unique microRNA expression patterns (Fig. 2D, E). By further comparing the abundantly expressed microRNAs of OPLL- and PLL-derived sEVs to their secreted hosts, we found that both OPLL- and PLL-derived sEVs displayed significant differences in microRNA expression patterns compared to their hosts (Fig. 2F, G). More strikingly, while OPLL and PLL cellular microRNA expression patterns exhibited some similarities, for instance, miR-21-5p and miR-125b-5p shared almost the same expression percentage in both cell lines, the EVs were overall very different (Fig. 2D–G). Furthermore, by comparing the differentially expressed microRNAs between OPLL and PLL, we found that the most differentially expressed microRNAs in sEVs exhibited negligible differences at the cellular level (Fig. 2H, I). Taking these results into consideration, we suspect that the microRNAs in OPLL or PLL sEVs may be actively selected for secretion into the microenvironment, which may play a vital role in the development of OPLL. To validate this hypothesis, we selected the top differentially expressed signal, miR-320e, as an example of OPLL-sEV-specific microRNAs for further study.

### MiR-320e is highly expressed in OPLL-sEVs and shows elevated expression during osteogenic induction

**Fig. 3:**
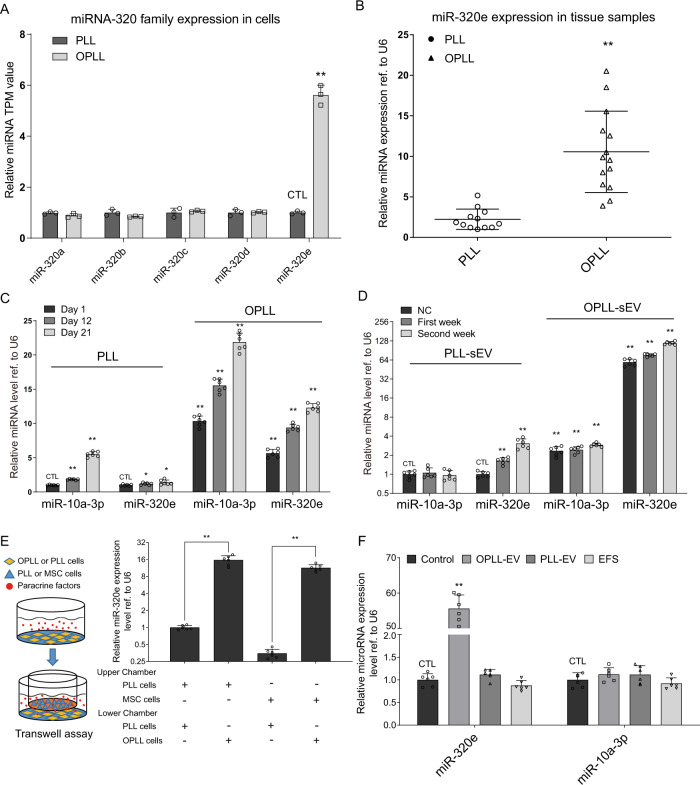
miR-320e is highly expressed in OPLL-sEV and upregulated in OPLL. **A** Comparison of the miRNA expression levels of miR-320 family in OPLL and PLL cell derived from High through-put sequencing data (*n* = 3, two tailed *t* test). TPM represents transcripts per kilobase million in the sequencing data. **B** qRT-PCR analysis detecting the expression level of miR-320e in OPLL (*n* = 14) and PLL (*n* = 12) tissues (two tailed *t* test). **C** qRT-PCR analysis detecting the expression level of miR-320e in osteogenic induced OPLL and PLL cells at different time point. Data showing respective microRNA expression fold changes compared to that of non-induced PLL cells (day 1) were presented (*n* = 6, two tailed *t* test). **D** qRT-PCR analysis detecting the expression level of miR-320e in osteogenic induced OPLL and PLL cell derived sEVs at different time point. Data showing respective microRNA expression fold changes compared to that of non-induced PLL cells derived sEV (NC) were presented (*n* = 6, two tailed *t* test). **E** Transwell assay followed by qRT-PCR analysis detecting the expression level of miR-320e in the co-cultured MSC or PLL cells (*n* = 6, two tailed *t* test). **F** qRT-PCR analysis detecting the expression level of miR-320e in sEV or EFS treated PLL cells (*n* = 6, two tailed *t* test). EFS represents with the supernatant of sEV collection that is free of sEVs. U6 level were detected and served as internal reference. All data were presented as the mean ± SD. *p < 0.05, **p < 0.01. Detailed statistical data and source data are provided in a Source Data file.

To fully reveal the characteristics of miR-320e, we first examined the expression level of the miR-320 family in PLL and OPLL cells using existing sequencing data. We found that only miR-320e exhibited a significant increase in expression in OPLL cells compared to PLL cells (Fig. 3A). Using OPLL and PLL patient tissue samples, we also confirmed a 5-6-fold increase in the expression of miR-320e (Fig. 3B). During the osteogenic induction of PLL and OPLL cells, we found that expression of miR-320e became gradually elevated in OPLL cells but was less significant than miR-10a-3p, a previously discovered OPLL-promoting microRNA in OPLL cells^6^ (Fig. 3C). However, in the sEVs of osteogenic-induced OPLL and PLL cells, expression of miR-320e was dramatically elevated compared to that of PLL and miR-10a-3p (Fig. 3D, S2B), which further confirmed miR-320e as an OPLL-sEV-specific microRNA. To provide evidence that miR-320e can be transmitted through sEVs, we performed a Transwell-assisted coculture experiment, and using qRT-PCR analysis, we found that cells cocultured with OPLL displayed increased miR-320e expression compared to controls (Fig. 3E). Direct addition of OPLL cell-derived sEVs also significantly increased the cellular levels of miR-320e (Fig. 3F). Overall, we confirmed miR-320e as an OPLL-sEV-specific and osteogenic-driven microRNA that can be transmitted through sEV uptake.

### MiRNA-320e actively modulates osteoblast differentiation of MSCs and ligament cells

**Fig. 4:**
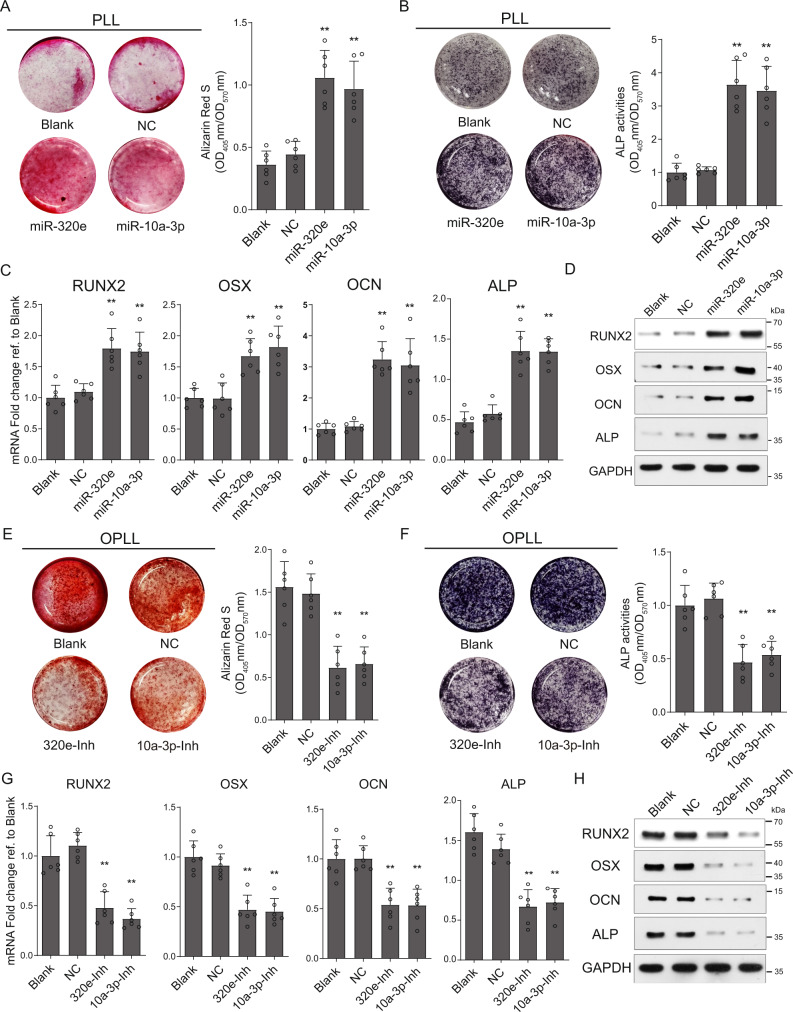
miR-320e promote the osteoblastogenesis of posterior longitudinal ligament cells. The osteogenic properties of PLL cells are analyzed using alizarin red staining (**A**) or alkaline phosphatase staining (**B**) after osteogenic induction for 21 days, *n* = 6. The colorimetric quantification is shown in the right panels, respectively (compared to NC group using two tailed *t* test). NC group represents transfecting scramble control miRNA mimics. The quantification of expression of ossification related genes were detected using either qRT-PCR (**C**, *n* = 6, compared to NC group using two tailed *t* test) or Western blot (**D**, *n* = 3 biologically independent repeats with similar results) under the same condition. Alizarin red staining (**E**) or alkaline phosphatase staining (**F**) were used to analysis osteogenic properties of miR-320e inhibition (320e-Inh) and miR-10a-3p inhibition (10a-3p-Inh) in OPLL cells after osteogenic induction for 21 days, *n* = 6. The colorimetric quantification is shown in the right panels, respectively (compared to NC group using two tailed *t* test). NC group represents transfecting scramble control miRNA mimics. Ossification-related genes are assessed by real-time PCR (**G**, *n* = 6, compared to NC group using two tailed *t* test) and Western Blot (**H**, *n* = 3 biologically independent repeats with similar results) after osteo-induction for 21 days under the same conditions respectively. GAPDH level were detected and served as internal reference. All data were presented as the mean ± SD. *p < 0.05, **p < 0.01. Detailed statistical data and source data are provided in a Source Data file.

In our previous studies, we demonstrated that OPLL cell-specific microRNA-10a-3p promotes osteogenic differentiation of PLL and OPLL cells into osteoblasts or their precursors^6^. Here, we overexpressed miR-320e mimics in PLL cells and examined their osteogenic ability. Using Alizarin red and ALP staining and quantification, we found that miR-320e-overexpressing PLL ligament cells exhibited enhanced ALP activity and greater calcium deposition after osteogenic induction (Fig. 4A, B), which was equivalent or more drastic than miR-10a-3p overexpression, especially with respect to calcium deposition. Upregulation of the expression of osteogenesis-related genes (*Runx2, Alp, Ocn, Osx*) was also confirmed by both qRT-PCR and western blot analysis in miR-320e-overexpressing PLL cells (Fig. 4C, D). Since miR-320e exhibited a relatively high abundance in the cellular level of OPLL cells, we chose to inhibit miR-320e in OPLL cells to examine the effect of miR-320e inhibition on osteogenesis. Using miR-320e- or miR-10a-3p-specific modified miRNA antisense inhibitors (antagomirs), we inhibited their expression and function in OPLL cells and observed significantly reduced Alizarin red staining and ALP activity in response to miR-320e inhibition in OPLL cells (Fig. 4E, F). Similar results were found in qRT-PCR and western blot analysis detecting expression changes of RUNX2, OSX, OPN and ALP (Fig. 4G, H). Since reports have shown that the development of OPLL may also be due to the osteogenic differentiation of MSCs^28,29^, we further evaluated the osteogenic promotive function of miR-320e on human bone marrow MSCs. As expected, similar results were observed in the Alizarin red staining, ALP activity assay and related gene expressions in osteogenic-induced MSCs (Fig. S3A-C). Moreover, by testing the effect of miR-320e in chondrogenesis of MSCs, we found that miR-320e overexpression significantly inhibited the chondrogenic differentiation of MSCs and its inhibition showed the opposite effect (Fig. S4A-C). In conclusion, we found that miR-320e actively promotes the osteogenic differentiation of ligament cells and MSCs in vitro.

### MiR-320e inhibits osteoclast formation in vitro

**Fig. 5:**
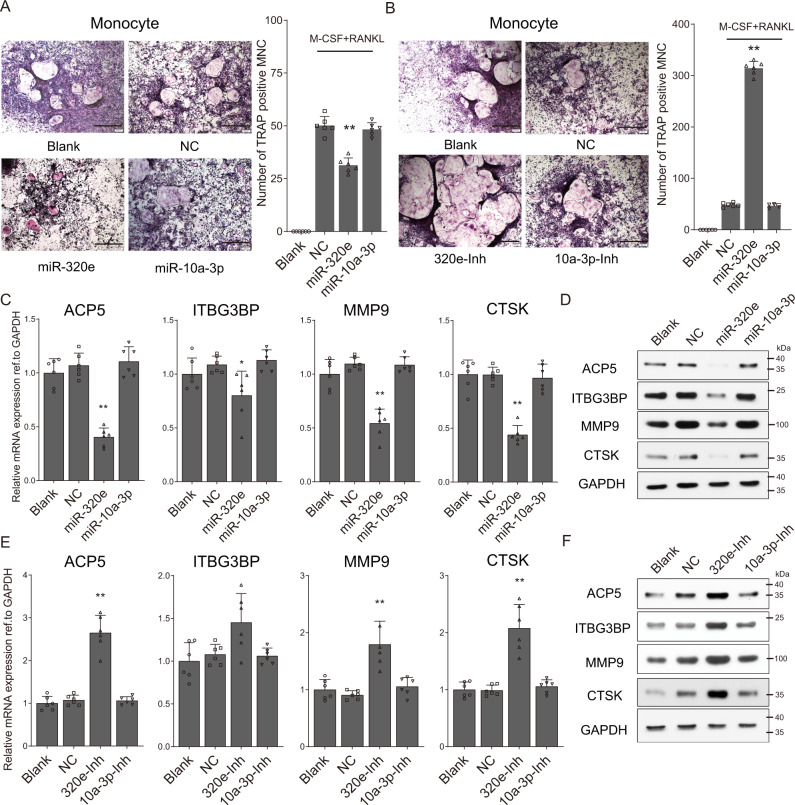
miR-320e inhibits the osteoclastogenesis of monocytes. Osteoclast differentiation was induced by treating the human monocyte cells with 30 ng/ml M-CSF and 100 ng/ml RANKL. Cells were fixed and stained for Tartrate-resistant acid phosphatase (TRAP) activities at days 20 in miR-320e and miR-10a-3p overexpression (**A**) and inhibition groups (**B**), *n* = 6, the scale bars represent 500 μm. The quantification of osteoclast precursors is shown in the right panel, respectively (compared to NC group using two tailed *t* test). The quantification of expression of osteoclastogenesis related genes were detected using either qRT-PCR (**C**, *n* = 6, compared to NC group using two tailed *t* test) or Western blot (**D**, *n* = 3 biologically independent repeats with similar results) in in miR-320e and miR-10a-3p overexpressed monocytes. Similarly, the quantification of expression of osteoclastogenesis related genes were detected using either qRT-PCR (**E**, *n* = 6, compared to NC group using two tailed *t* test) or Western blot (**F**, *n* = 3 biologically independent repeats with similar results) in in miR-320e and miR-10a-3p inhibited monocytes. GAPDH level were detected and served as internal reference. All data were presented as the mean ± SD. *p < 0.05, **p < 0.01. Detailed statistical data and source data are provided in a Source Data file.

The normal bone formation process maintains a dynamic osteoblastogenesis-osteoclastogenesis balance. However, the bone mass formed in OPLL was reported to be more similar to dystrophic calcification-based bony metaplasia^30^. Since the effect of osteoblastogenesis was evaluated, we hypothesized that miR-320e may disrupt normal osteoclastogenesis to promote metaplasia in the ligament tissue. We found that overexpression of miR-320e in human monocytes significantly increased the formation of osteoclasts or precursors, as observed by tartrate-resistant acid phosphatase (TRAP) staining (Fig. 5A), while miR-320e inhibition showed the opposite results (Fig. 5B). Moreover, qRT-PCR and western blot analysis detecting osteoclastogenesis-related genes revealed a significant increase in the miR-320e group (Fig. 5C, D) but a significant decrease in the miR-320e inhibition group (Fig. 5E, F). Collectively, these results indicate the possibility that miR-320e may play dual roles in regulating both osteoblastogenesis and osteoclastogenesis in the development of OPLL. Interestingly, no significant differences were found in the miR-10a-3p or NC group, indicating that the effect on osteoclast formation might be miR-320e-specific.

### TAK1 is targeted by miR-320e in OPLL

**Fig. 6:**
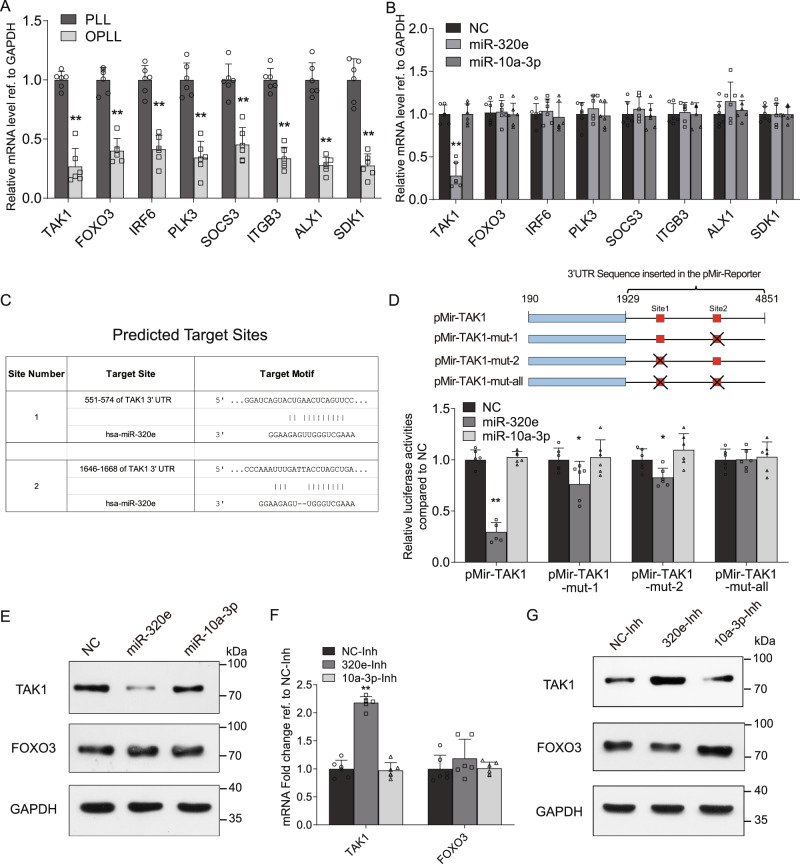
TAK1 is targeted by miR-320e. **A** qRT-PCR analysis detecting the mRNA expression levels of miR-320e candidate targets in PLL and OPLL cells, *n* = 6, two tailed *t* test. **B** qRT-PCR analysis detecting the mRNA expression levels of miR-320e predicted targets after miR-320e or miR-10a-3p overexpression in PLL cells, *n* = 6, two tailed *t* test. Here miR-10a-3p is served as a negative miRNA control that are not correlated with the candidates. **C** MiRanda prediction of miR-320e binding motif in TAK1 3’UTR. Note that the sites were picked according to the binding energy and rodent conservation. **D** Dual luciferase reporter assay detecting the activities of firefly luciferase generated by respective 3’UTR bearing plasmids after miR-320e or miR-10a-3p overexpression in HEK293T cells (*n* = 6, two tailed *t* test). **E** Western Blot analysis showing the protein levels of TAK1 or FOXO3 after miR-320e or miR-10a-3p overexpression in PLL cells. Here, FOXO3 is served as a negative target control, which miR-320e had no function on it, *n* = 3 biologically independent repeats with similar results. **F** mRNA levels of TAK1 and FOXO3 after miR-320e or miR-10a-3p inhibition in OPLL cells using qRT-PCR analysis, *n* = 6, two tailed *t* test. **G** Western Blot analysis showing the protein levels of TAK1 or FOXO3 after inhibition of miR-320e or miR-10a-3p in OPLL cells, *n* = 3 biologically independent repeats with similar results. GAPDH level were detected and served as internal reference. All data were presented as the mean ± SD. *p < 0.05, **p < 0.01. Detailed statistical data and source data are provided in a Source Data file.

To explore the underlying mechanism of miR-320e, we took advantage of our previous OPLL and PLL cellular transcriptome data and miRanda miRNA target prediction algorithm. We screened for ossification-related candidates and found that 8 potential targets were significantly decreased in OPLL cells (Fig. 6A). Using miRNA mimic overexpression in PLL cells, we found that only the expression of TAK1 was significantly reduced (Fig. 6B). Together, we presumed that TAK1 may be a target of miR-320e in OPLL cells. To further confirm the relationship between miR-320e and TAK1, we performed a luciferase reporter assay based on the miRanda algorithm to predict conserved target sites (Fig. 6C). We constructed a luciferase reporter plasmid encoding the wild type 3’UTR of TAK1 and various target site mutated reporter plasmids and transfected them with miR-320e into HEK-293T cells to verify their targeting relationship. Using a dual luciferase reporter assay, we found that luciferase activities were significantly downregulated in response to miR-320e overexpression, and this effect was attenuated in the mutated group, with no significant reduction in luciferase activities in the group with all sites mutated (Fig. 6D). Furthermore, western blot analysis was performed to confirm the inhibitory effect of miR-320e on TAK1 at the protein level (Fig. 6E). To provide more evidence, we inhibited miR-320e expression in OPLL cells and found that expression of TAK1 but not FOXO3 was significantly upregulated at both the mRNA and protein levels (Fig. 6F, G). Taken together, these results demonstrate that miR-320 directly binds TAK1 mRNA and reduces its expression at both the RNA and protein levels.

### MiR-320e suppresses TAK1 to regulate osteoblastogenesis and osteoclastogenesis

**Fig. 7:**
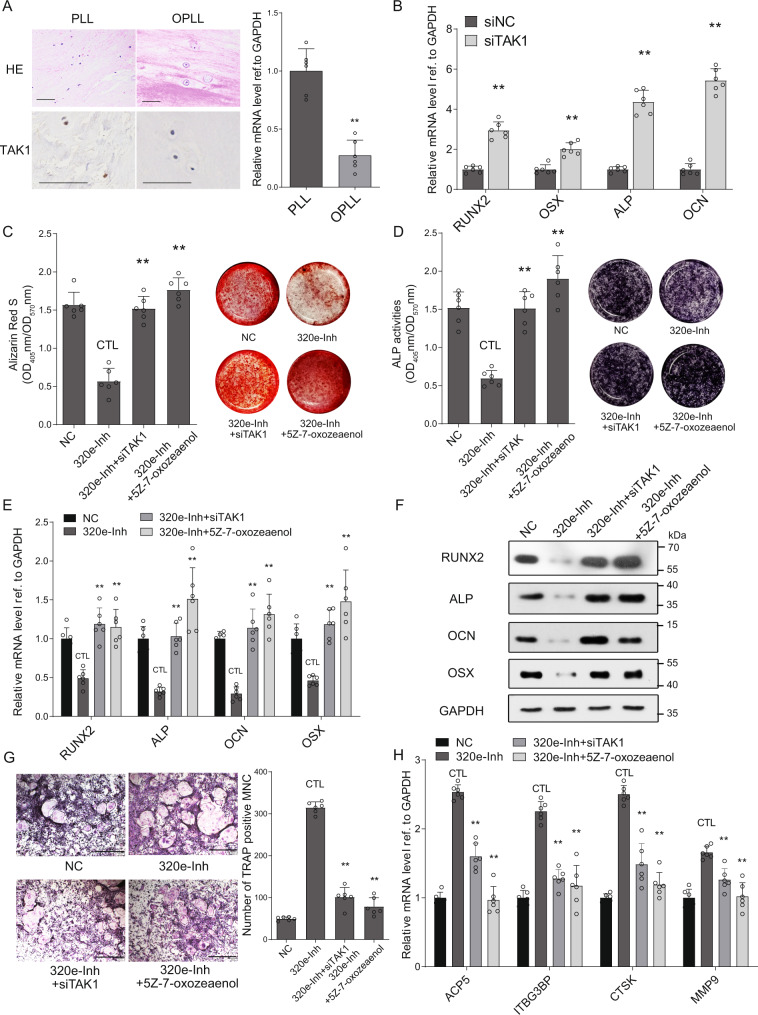
TAK1 is essential for miR-320e to regulate osteoblastogenesis and osteoclastogenesis. **A** In situ hybridization and histochemistry is used to analysis the expression level of TAK1 in PLL and OPLL patients’ tissue (*n* = 6, two tailed *t* test, the scale bars represent 500 μm). **B** qRT-PCR analysis showing the mRNA expression level of osteogenic genes after knockdown of TAK1 using small interference RNAs in osteogenic induced PLL cells, *n* = 6, two tailed *t* test. The siNC represents transfecting scramble control siRNAs which serve as control group. Alizarin red staining (**C**) or alkaline phosphatase staining (**D**) was used to analysis osteogenic properties of respective treatment in miR-320e inhibited and osteogenic induced OPLL cells after various treatment, both *n* = 6, two tailed *t* test. The colorimetric quantification is shown in the right panels, respectively. 5Z-7-oxozeaenol (a specific inhibitor of TAK1) is used at 20 nM to inhibit TAK1 activities. The relative RNA level (**E**, *n* = 6, two tailed *t* test) and protein level (**F**, *n* = 3 biologically independent repeats with similar results) of osteogenic genes in miR-320e inhibited and osteogenic induced OPLL cells after various treatment are shown. **G** Osteoclast induction combined with TRAP staining was used to evaluate the function of miR-320e inhibition and TAK1 inhibition in monocytes, the scale bars represent 500 μm. The quantification of osteoclast precursors is shown in the right panel, *n* = 6, two tailed *t* test. The quantification of expression of osteoclastogenesis related genes were detected using qRT-PCR (**H**) under the same treatment in monocytes, *n* = 6, two tailed *t* test. GAPDH level were detected and served as internal reference. All data were presented as the mean ± SD. **p < 0.01. Detailed statistical data and source data are provided in a Source Data file.

We first examined expression of TAK1 at the tissue level. As expected, the expression of TAK1 was significantly downregulated in OPLL tissues but highly expressed in PLL tissues by immunohistochemistry analysis (Fig. 7A, left panel) and qRT-PCR analysis (Fig. 7A, right panel). By using TAK1-specific siRNAs (Fig. S5A), we found that the expression of osteogenic genes was upregulated after TAK1 knockdown in PLL cells (Fig. 7B). Using Alizarin red and ALP staining and quantification, we also found that TAK1 knockdown or inhibition using 5Z-7-oxozeaenol (a TAK1-specific inhibitor) significantly upregulated ALP activities and calcium deposition in OPLL cells after osteogenic induction (Fig. S5B, C). In the osteoclast differentiation assay, the number of differentiated osteoclasts or their precursors was decreased significantly (Fig. S5D).

To determine the importance of TAK1 in the miR-320e-mediated osteogenic promotion effect, we performed a rescue assay using Alizarin red and ALP staining in osteogenic-induced OPLL cells. The results showed that knockdown of TAK1 or inhibition using 5Z-7-oxozeaenol significantly reversed the decreased mineral deposition and ALP activities caused by miR-320e inhibition (Fig. 7C, D). Further analysis confirmed a similar rescue effect of TAK1 knockdown or inhibition on the expression change of osteogenesis-related genes at both the RNA and protein levels (Fig. 7E, F). In the osteoclast differentiation assay, we found that knockdown or inhibition of TAK1 also significantly reversed the increased osteoclast formation numbers in the miR-320e-inhibited group compared to the other groups (Fig. 7G). Additionally, osteoclastogenesis-related genes displayed similar effects after TAK1 knockdown or inhibition in miR-320e-inhibited monocytes (Fig. 7H, S5E). Further TAK1 3’UTR overexpression assay is also performed in miR-320e overexpressed PLL and monocytes, and similar results were observed (Fig. S6A-D), which confirmed that the function of miR-320e requires binding to the TAK1 3’UTR to affect the downstream function of TAK1. Since the common downstream pathways of TAK1 involve NF-κB and MAPK signaling, we assessed whether an NF-κB inhibitor (SC75741) or MAPK p38 inhibitor (SB203580) could reverse the effect of miR-320e inhibition in MSCs, ligament cells and monocytes. By using qRT-PCR analysis, we found that only NF-κB inhibition significantly rescued the phenotypic change of miR-320e in MSCs, ligament cells and monocytes (Fig. S7A–C). Taken together, these results indicate that TAK1 is necessary for miR-320e to modulate osteoblastogenesis and osteoclastogenesis in vitro.

### MiR-320e is required for OPLL-derived sEVs to promote osteogenesis in vitro

**Fig. 8:**
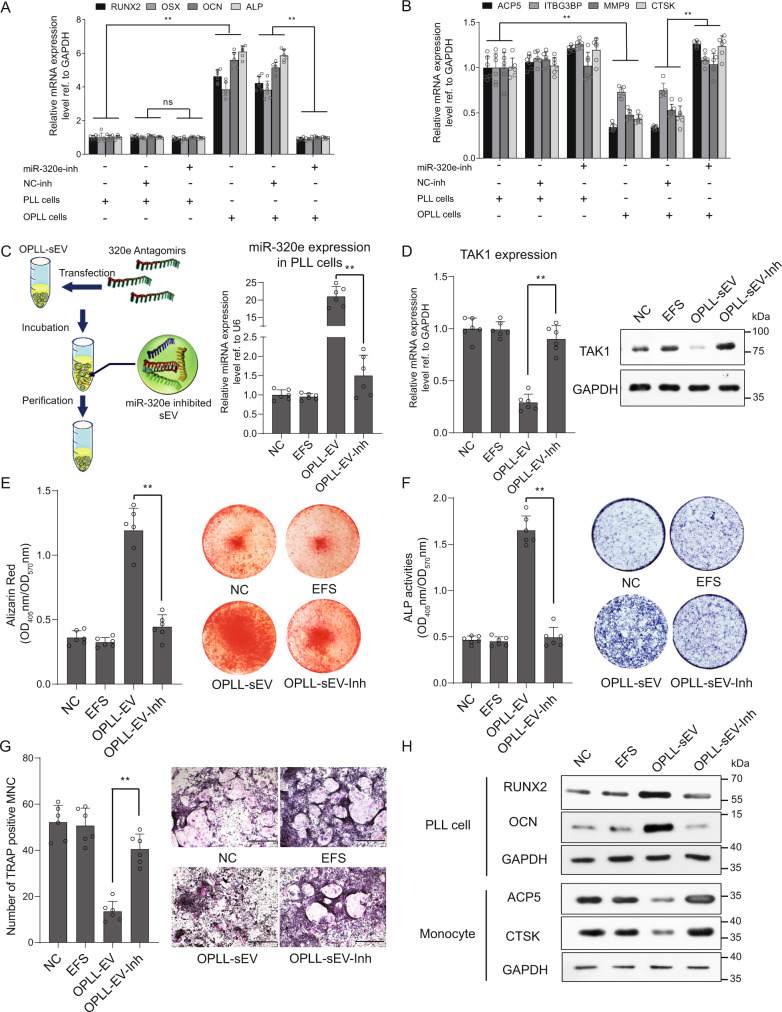
miR-320e is needed for OPLL derived sEVs to modulate osteoblastogenesis and osteoclastogenesis. Transwell assay followed by qRT-PCR analysis detecting the expression changes of osteogenic related genes (**A**) or osteoclastogenic related genes (**B**) in PLL cells (**A**) or Monocyte (**B**) co-cultured with PLL cells or OPLL cells treated with miR-320e inhibitor or NC, *n* = 6, two-way ANOVA. **C** scheme of generation of miR-320e inhibited sEVs. The efficiency of inhibition is detected using qRT-PCR analysis in PLL cells shown in the right panel, *n* = 6, two tailed *t* test. **D** The effect of miR-320e inhibited sEVs on TAK1 expression detected by qRT-PCR (*n* = 6, two tailed *t* test) or Western blot (*n* = 3). OPLL-sEV-Inh represents miR-320e inhibited OPLL derived sEVs. The osteoblastogenic properties were examined by alizarin red staining (**E**) or alkaline phosphatase staining (**F**) and quantification in osteogenic induced PLL cells, *n* = 6, two tailed *t* test. **G** The osteoclastogenic properties were examined by osteoclast induction combined with TRAP staining and quantification, *n* = 6, two tailed *t* test, the scale bars represent 500 μm. **H** The relative protein level of osteoblastogenesis or osteoclastogenesis related genes were detected using Western blot (*n* = 3 biologically independent repeats with similar results). GAPDH level or U6 level were detected and served as internal reference. All data were presented as the mean ± SD. **p < 0.01. Detailed statistical data and source data are provided in a Source Data file.

Here, we performed qRT-PCR analysis to reveal the effect of miR-320e inhibition in PLL and monocytes cocultured with OPLL or PLL cells during osteogenic or osteoclastogenic differentiation. The results revealed that osteogenic genes were significantly upregulated when cocultured with OPLL cells, but this effect was reversed after miR-320e inhibition (Fig. 8A). Similar results were observed in monocytes cocultured with OPLL cells (Fig. 8B). To obtain direct evidence on the importance of miR-320e to OPLL-sEVs, we modified OPLL-derived sEVs by transfection of miR-320e inhibitor into the collected sEVs (Fig. 8C), and initial qRT-PCR analysis showed decreased miR-320e expression in miR-320e-inhibited-sEV treated PLL cells compared to normal OPLL-sEV group (Fig. 8C). By detecting the RNA and protein levels of TAK1 in these PLL cells, we found that miR-320e-inhibited sEVs (OPLL-sEV-Inh) exerted no significant effect on TAK1 expression, while wild type normal OPLL-sEV significantly decreased the expression of TAK1 (Fig. 8D). Further Alizarin red and ALP staining and quantification in osteogenic-induced PLL cells revealed that miR-320e-inhibited sEVs significantly reversed the osteogenic promotion effect of OPLL-sEVs (Fig. 8E, F). In the osteoclast differentiation assay, miR-320e-inhibited sEV treatment induced increased TRAP-positive osteoclast cells than OPLL-sEVs (Fig. 8G). These findings were further supported by western blot analysis confirming that miR-320e-inhibited sEVs had the opposite effect as OPLL-sEVs on osteogenic and osteoclastogenic genes (Fig. 8H). Overall, we demonstrated that OPLL-derived sEVs promote ligament cell ossification partly through sEV-specific miR-320e.

### OPLL-sEVs promote ossification by transmitting miR-320e in vivo

**Fig. 9:**
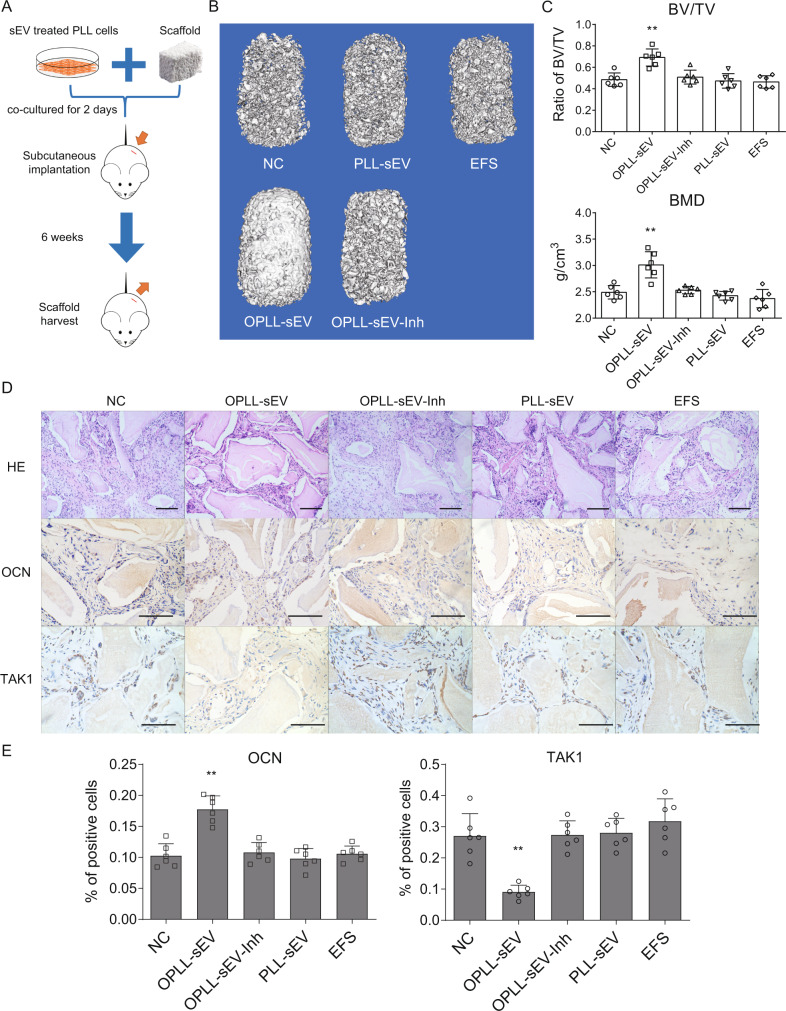
miR-320e promoted heterotopic bone formation in vivo. **A** Scheme for heterotopic bone formation assay procedure. Small EVs (sEV) were treated with OPLL cells prior to implantation. **B** Representative reconstructed three-dimensional micro-CT images of implanted bio-scaffold after 6 weeks, *n* = 6 for each group. **C** Bone analysis showing the BV (bone volume)/TV (tissue volume) and BMD (bone marrow density) of cultured bone constructs (*n* = 6, one-way ANOVA). **D** H&E staining and immunohistochemical staining of implanted bio-scaffold after 6 weeks in respective groups, *n* = 6 for each group. The scale bars represent 500 μm. **E** Quantification of OCN and TAK1 expression in the immunohistochemical staining of implanted bio-scaffold after 6 weeks (*n* = 6, one-way ANOVA). All data were presented as the mean ± SD. *p < 0.05, **p < 0.01. Detailed statistical data and source data are provided in a Source Data file.

To further analyse the function of miR-320e in vivo, we performed a heterotopic bone formation assay in nude mice. PLL cells were treated with OPLL- or PLL-derived sEVs and cocultured with Bio-Oss Collagen scaffolds for 2 days in osteogenic induction medium. The scaffolds and seeded cells were then implanted into the subcutaneous region on the backs of nude mice (*n* = 6) and allowed to grow for 6 weeks (Fig. 9A). Micro-CT was used to detect the bone mass and the bone volume of the scaffolds after sacrifice, and improved bone morphology was observed in the 3D reconstructed scaffold image of the OPLL-sEV group, while the miR-320e inhibited group (OPLL-sEV-Inh) exhibited less bone mass formation than the OPLL-sEV group (Fig. 9B). The ratio of bone volume/tissue volume (BV/TV) and bone mineral density (BMD) were significantly increased in the OPLL-sEV-treated group, and miR-320e inhibition reversed this effect (Fig. 9C). Further histological examination showed that the OPLL-sEV-treated group formed more bone tissue, while the miR-320e inhibition group formed less bone tissue (Fig. 9D). Immunohistochemistry analysis of OCN and TAK1 also showed similar results: the OPLL-sEV-treated group had more OCN-positive cells and fewer TAK1-positive cells in the bone mass, while the miR-320e inhibition group showed fewer OCN-positive cells (Fig. 9D, E). Together, these results indicate that OPLL-derived sEVs promote ossification in vivo, the effect of which is largely due to miR-320e.

### OPLL-EVs promote OPLL formation via miR-320e in vivo

**Fig. 10:**
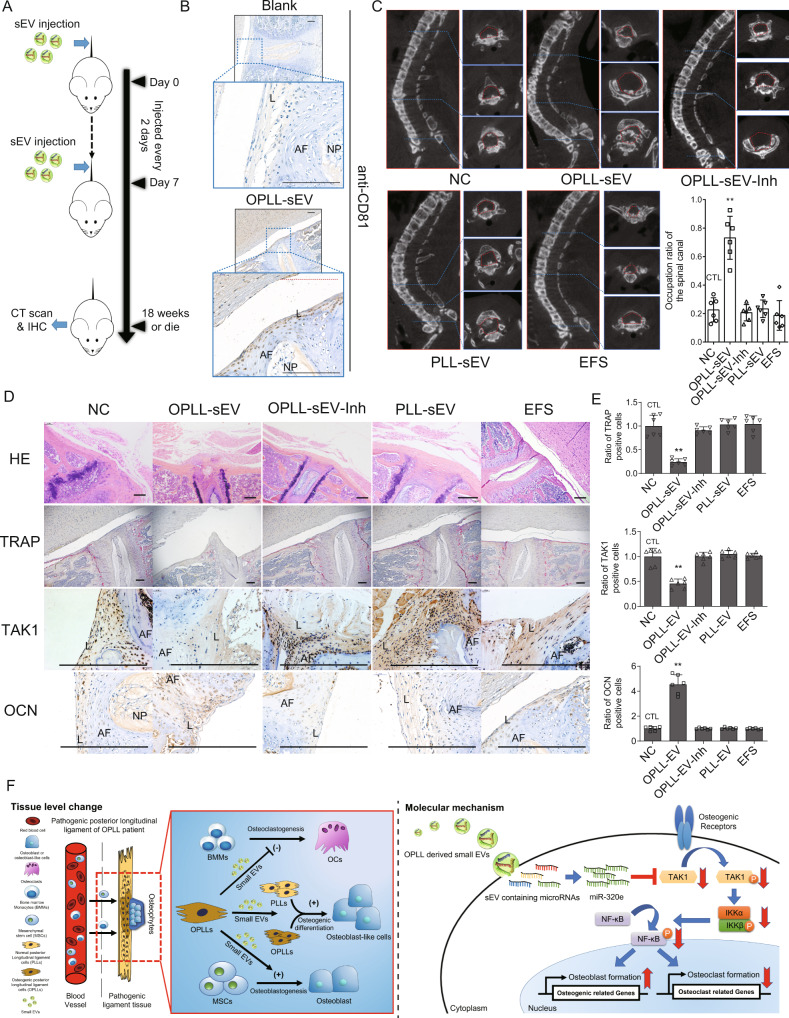
sEV derived miR-320e promoted OPLL development in ttw mice. **A** Experimental outline of in vivo OPLL formation assay using *ttw* mice injected with OPLL-sEVs or miR-320e inhibited sEV. Injections were performed once every 2 days for one week. Mice were sacrificed upon death or until 18 weeks after last injection, spine samples were harvested and limb functions were observed everyday. **B** Immunohistochemistry analysis showing the distribution of human CD81 expression in OPLL-sEV injected spine tissue (*n* = 3). Note that in the Blank group, no cellular CD81 expression were found. While in OPLL-sEV injected group, the ligament cells displayed strong expression of human CD81. The scale bars represent 500 μm. **C** micro-CT images of spine harvested spines from *ttw* mice in various groups. The occupation percentage of the ossified mass in the spinal canal of *ttw* mice were compared (lower right panel, *n* = 6, one-way ANOVA). **D** H&E staining, TRAP staining and immunohistochemistry analysis showing the expression of TAK1 and OCN in the spine samples from treated *ttw* mice were analyzed (*n* = 6). AF stands for annulus fibrosus, NP stands for nucleus pulposus and L stands for posterior longitudinal ligament. The scale bars represent 800 μm. **E** The quantification of TRAP positive, TAK1 positive and OCN positive ligament cells in the posterior longitudinal ligament region were shown and compared, *n* = 6 for each marker tested, one-way ANOVA. **F** The conclusive illustrations depict the potential mechanism uncovered in this study. All data were presented as the mean ± SD. *p < 0.05, **p < 0.01. Detailed statistical data and source data are provided in a Source Data file.

Despite these findings, the effect of OPLL-derived sEVs or miR-320e in OPLL disease has not been elucidated. Therefore, taking advantage of *ttw* (tiptoe walking, or tiptoe walking Yoshimura) OPLL disease model mice, we performed systematic injection of sEVs or miR-320e-inhibited sEVs to investigate their effect in vivo (Fig. 10A). Initial immunohistochemistry analysis of EV markers was performed in the spinal ligament area of day 7 mice to show that injected small EVs could successfully arrive and absorbed by the ligament cells. Results showed that compared to control group which injected with water, the OPLL-EV injected mice showed significant increase in the expression of human CD81 and CD63, which indicated that human OPLL-EV could be abundantly absorbed by ligament cells compared to intervertebral cells in the spine tissue (Fig. 10B, Fig. S8A). Micro-CT was used to analyse the presence and occupation percentage of ossified ligament mass in the spine canal of the mice. The results showed that OPLL-sEV-injected mice displayed larger osteophytes and increased spinal canal occupation in the posterior longitudinal ligament region, while miR-320e-inhibited-sEV treated mice did not exhibit significant differences in osteophyte size or spinal canal occupation rate compared to control groups (Fig. 10B, C). Here, we observed limb spasms during movement, weakness in the limbs and abnormal gait in *ttw* mice and defined them as neurological symptoms (Supplementary Videos 1 & 2), and found OPLL-EV treated mice manifest neurological symptoms earlier than other groups. (Fig. S8B). In addition, we observed early death that shifted the survival curve in the OPLL-EV-treated group, while death occurred at approximately 18 weeks of age in the other groups, as expected (Fig. S8A). Histochemistry analysis revealed thickened and ossified ligaments, increased OCN level, lowered TAK1 expression level and fewer TRAP-positive cells were found in the OPLL-sEV-treated group, while other groups exhibited no significant differences (Fig. 10D, E). Consistent with our former findings, we demonstrated that OPLL cell-secreted sEVs promote the osteogenesis and inhibit osteoclastogenesis both in vivo and in vitro, the mechanism of which may be partly due to miR-320e transmission (Fig. 10F). Our findings provide initial evidence that OPLL cells modulate the disease-specific microenvironment through small EV secretion.

## Discussion

OPLL is a hyperostotic disease of the ligament of the spine, where the posterior longitudinal ligament (PLL) becomes progressively ossified, often leading to symptomatic spinal canal stenosis. Genetic, environmental, and biochemical factors have been implicated in the development of this disease with a high prevalence in the Asian population. From an aetiological perspective, OPLL can be divided into two categories: primary (idiopathic) OPLL or secondary (syndromic) OPLL. The latter includes OPLL associated with genetic diseases, such as hypophosphatemic rickets, osteomalacia or diffuse idiopathic skeletal hyperostosis (DISH). With advances in diagnostic technologies, the incidence of OPLL, especially among older people over 65 years of age, has reached more than 20%^1^. Recent reports have shown an increasing incidence of OPLL in European and other Western populations^31,32^, indicating that OPLL is becoming a global health problem that requires more attention. However, treatment of OPLL is sometimes unsatisfactory. Since early-stage OPLL patients usually do not display neurological symptoms, many symptomatic patients have a high risk in surgery due to the large osteophytes that occupy most spaces of the spinal canal and are prone to serious complications, such as root nerve palsy or spinal cord injury. Until now, no efficient therapeutic strategy, particularly with respect to pharmacotherapy or preventive intervention of OPLL, has been elucidated or suggested due to the poor understanding of the molecular mechanism of this disease, leaving surgical management as the only option for symptomatic OPLL patients. Thus, gaining more insight and understanding of the pathological mechanism of the disease is needed to further develop nonsurgical treatment approaches.

Many reports on the underlying mechanisms of OPLL have suggested that OPLL is a multifactorial disease influenced by genetic and environmental (nongenetic) factors^33^. Since many of these genetic variations and association studies vary greatly from each other and no direct evidence has shown how these factors are linked to the onset or progression of the disease, much attention should be given to uncovering the regulatory mechanism and more common factors to provide a basis for further understanding the disease. Studies have shown that extracellular vesicles (EVs) are critical microenvironmental factors that directly affect the development of various diseases. Disease-related EVs could strongly influence the proliferation or differentiation ability of cells^34^. These small vesicles, ranging between 30–150 nm in size, carry important signaling proteins, lipids and nucleic acids, which are transmitted by the uptake of vesicles of other cells. Both EV-containing proteins and RNAs are vital players in many biological or pathological processes. During our previous studies, we identified many OPLL disease-specific cellular miRNAs that could shape the disease regulatory network that mediates the expression of many ossification-related genes^6,35^. Although identified as upstream factors of the disease, how these miRNAs are regulated in normal ligament cells is less well understood. On this basis, we aimed to determine how pathogenic cells influence the microenvironment of the posterior longitudinal ligament in this study. Our data demonstrated that pathogenic OPLL cells secrete small extracellular vesicles (sEVs) to affect the osteogenic properties of normal ligament cells and mesenchymal stem cells, the mechanism of which involves transmission of OPLL-specific miR-320e in vitro. By using the *ttw* OPLL mouse model, we also showed that OPLL derived sEVs were capable of promoting ossification in vivo. Together, these data provide an important basis for the notion that pathogenic ligament cells can modify the microenvironment towards an ossification-favouring condition via sEV secretion.

Osteogenesis is a synergistic event involving various cell types, such as osteoblasts and osteoclasts. In OPLL, although no study has been dedicated to identifying what cell types may take part in disease development, there are studies that claim mesenchymal stem cells (MSCs) or the ligament cells themselves are responsible for the ossification process^6,36^. From a histological perspective, the posterior longitudinal ligament is composed of layers of fibroblast-like ligament cells^37^. During the early stages of the disease, the ligament becomes atrophied, and calcified foci begin to develop. During this time, increased blood supply around the ligament often develops. As the disease progresses, calcified foci became bony structures that are isolated from the vertebrae, exhibiting a unique “double layer” sign on computed tomography. Whether the bone formation process in the ligament occurs through endochondral ossification, extension of the existing bone, or the dystrophic calcification-bony metaplasia sequence is still under debate^30^. However, in our previous studies, we provided evidence that OPLL patient-derived posterior longitudinal ligament cells can be differentiated into osteoblasts and are more prone to osteoblast differentiation than normal ligament cells^6^. Nevertheless, we considered all ossification-related cells, including MSCs, ligament cells and even monocytes (osteoclast precursor cells), and examined the effect of OPLL-derived sEVs on these cells. During our research, we found that ligament cells, MSCs and monocytes are all affected by OPLL-derived sEVs during osteoblast or osteoclast differentiation in vitro, indicating that OPLL ligaments are more prone to ossification.

The development of OPLL involves the regulation of osteogenesis by disease-specific miRNAs. In previous studies, we identified many OPLL-specific miRNAs that may contribute to the osteogenic process, and the mechanism of some of them was unveiled. The miRNA we identified in our study, miR-320e, is also a highly upregulated microRNA in OPLL cells; however, the abundance of miR-320e at the cellular level was only ~10 TPM in OPLL cells, which was ~30 times lower than that in secreted sEVs. The aggregated expression of miR-320e in sEVs indicated that functional disease-specific miRNAs were more clustered in sEVs than in cells, allowing them to be transmitted to other cells to exert their disease-promoting function. This unique aggregation of miRNAs can also be found in many other diseases, which further supports our finding that miR-320e may be an important regulator of OPLL pathological processes through its transmission to other normal cells to promote their ossification. In our research, the miR-320e we studied was only a typical example of such a function of OPLL-derived sEVs. We also found that many miRNAs, such as miR-6720-3p and miR-3185, had expression patterns similar to that of miR-320e and may also contribute to the development of OPLL through other mechanisms. However, the data we provided here show such a function in the OPLL disease setting.

By computational prediction, we found that TAK1 is the primary target of miR-320e and showed that OPLL-derived sEVs regulate monocyte osteoclast differentiation and ligament cell osteogenic differentiation through downregulation of TAK1. Transforming growth factor-beta-activated kinase-1 (TAK1) is a member of the mitogen-activated protein kinase kinase kinase (MAP3K) family with high sequence similarity to Raf-1 and MEKK-1^38^. It can be activated by RANKL and BMPs and has an important role in the MAPK signaling pathway. It has been reported to play an essential role in osteoclast and osteoblast differentiation. Knocking out TAK1 in monocytes causes impairment of osteoclast formation^39^, and a recent study reported that TAK1-deficient mice displayed osteopetrosis, providing further in vivo credibility^40^. For osteoblast differentiation, the function of TAK1 is rather intriguing. Some reports have shown that TAK1 knockdown promotes the osteoblastic differentiation of MSCs, but others have shown the opposite effect^41,42^. However, since p38 MAPK is critically involved in the osteogenic differentiation of MSCs, it is believed that TAK1 serves as a balancing factor that controls both the early and late stages of osteoblast differentiation^39^. In our study, we demonstrated that TAK1 was significantly downregulated in response to overexpression of miR-320e, promoted the osteogenic differentiation of human ligament cells, and significantly reduced the formation of osteoclasts from monocytes. This demonstrated that TAK1 is necessary for the osteoblastic differentiation of ligament cells in OPLL.

Overall, we have demonstrated that sEV-derived miRNAs are indeed vital regulators of the osteo-pathogenesis process in OPLL. However, how miR-320e initiates its upregulation is still unknown. Nevertheless, we provided both in vitro and in vivo evidence that OPLL patient’s spinal ligament cell-derived small EVs contribute to the development of OPLL by promoting the osteogenic differentiation of MSCs and ligaments while inhibiting osteoclast formation, the molecular mechanism of which is largely due to EV-transmitted miR-320e modulation of TAK1 signaling in both ligament cells and MSCs.

## Methods

### Sample collection and primary cell culture

All experimental protocols were approved by the Ethics Committee of the Naval Medical University. Ligament samples were obtained from each participant with written informed consent, and all related methods were performed in accordance with the approved guidelines. The diagnosis of OPLL or PLL (spinal trauma patients who underwent cervical corpectomy) was confirmed by computerized tomography (CT) and magnetic resonance imaging preoperatively in our institution. OPLL or PLL ligament specimens were obtained intraoperatively by isolating the soft ligament tissue of patients undergoing anterior cervical corpectomy and fusion surgery using an ultrasonic bone curette^43^ and were immediately processed for primary cell culture as previously described^6^. In brief, small pieces of ligament tissue were plated on culture dishes in Dulbecco’s modified Eagle’s medium (DMEM, Life Technology Gibco, USA) supplemented with 10% foetal bovine serum (FBS, Gibco, USA), 1% L-glutamine (Gibco, USA), and 1% penicillin/streptomycin (Gibco, USA) in a humidified atmosphere containing 95% air and 5% CO_2_ at 37 °C. The fibroblast-like cells that migrated were harvested for further culture, the culture medium was changed every two days, and cells were passaged using trypsin when they reached 90% confluence. Cells at passages 2 to 6 were used for further experimental analysis. Altogether, 16 OPLL patient tissue samples (11 males and 5 females, aged 47–65 years, mean age 53.8 years) and 12 PLL patient samples (7 males and 5 females, aged 48–64 years, mean age 54.2 years) were collected during anterior cervical corpectomy. General patient information is provided in Supplementary Data 1.

Human bone marrow mesenchymal stem cells were purchased from Cyagen Biosciences (Guangzhou, China). Adherent MSCs were cultured in flasks in hMSC growth medium (Cyagen Biosciences, Inc., Guangzhou, China) in an incubator at 37 °C with 5% CO_2_ and were passaged after reaching 80% confluence. Cells from passages 2–7 were used in subsequent experiments.

### Osteogenic, osteoclastogenic and chondrogenic differentiation

For osteogenic differentiation, MSCs and ligament cells from PLL or OPLL patients were seeded into 12-well or 24-well cell culture plates at a density of 1 × 10^4^/cm^2^ and incubated for 48 h at 37 °C under 5% CO_2_. After 24 h, the medium was changed to osteogenic induction medium (L-DMEM with 10% FBS, 100 IU/ml penicillin/streptomycin, 100 nM dexamethasone, 0.2 mM ascorbic acid, and 10 mM β-glycerophosphate). Cells were maintained for 21 days by refreshing the osteogenic induction medium every 3 days.

For chondrogenic differentiation, MSCs were seeded into 12-well or 24-well cell culture plates at a density of 5 × 10^5^ cells and incubated for 48 h at 37 °C under 5% CO_2_. The medium was subsequently changed to StemPro^®^ chondrogenic differentiation medium (Invitrogen, USA) for 21 days. All media were changed twice per week.

For monocyte differentiation, we first obtained monocytes from the blood of non-OPLL patients. Six samples were isolated and used in our study, and subjects ranged from 45 to 63 years of age (4 males and 2 females). The peripheral blood of patients was collected into heparinized tubes, diluted 1:1 in αMEM culture medium (Gibco, Paisley, UK), layered over Ficoll-Hypaque (Pharmacia, Milton Keynes, UK) and centrifuged at 700 × *g* for 20 min at 4 °C. The peripheral blood mononuclear cell (MNC) layer was removed and washed in αMEM, and the cell pellet was resuspended in αMEM/FBS medium. The number of MNCs in the cell suspension was counted using a haemocytometer after lysis of red cells with 5% v/v acetic acid solution. For osteoclast differentiation, monocytes were cultured in 24-well tissue culture plates containing 1 ml αMEM/FBS in the presence of RANKL (80 ng/ml, R&D Systems, Shanghai, China) and M-CSF (50 ng/ml, R&D Systems, Shanghai, China). All cultures were incubated for 14 days, and the culture medium containing these factors was replaced every 4 days.

### Isolation and identification of small EVs

Small EV (sEV) isolation and identification followed the MISEV 2018 guidelines^44^. sEVs were isolated from cell medium cultured with 2% EV depleted FBS before conditioned medium collection. Cells were collected by centrifugation at 500 × *g* for 5 min at 4 °C and the supernatant was stored at −20 °C until further use. sEVs were isolated by differential ultracentrifugation as previously described^24,25^. Briefly, cells were pelleted at 200 g for 5 min and supernatant was centrifuged at 2000 x *g* for 15 min to remove dead cells and again at 16,000 × *g* for 45 min to remove cell debris, and supernatant was then filtered through a 0.22-µm filter and the flow-through was transferred to new tubes and then centrifuged again at 120,000 g for 70 min at 4 °C in a SW32Ti rotor (Beckman Coulter, Inc., Pasadena, CA) to pellet the sEVs. The supernatant was then filtered through a 0.22-µm filter, and the flow-through was transferred to new tubes and then centrifuged again at 120,000 g for 70 min at 4 °C in a SW32Ti rotor (Beckman Coulter, Inc., Pasadena, CA) to pellet the sEVs. The supernatant was also stored in other tubes as EV-free supernatant (EFS). The pellet was washed with 25 ml of PBS (without calcium or magnesium ions and with 1 U/μl RNAse inhibitor) and centrifuged again at 120,000 g for 70 min at 4 °C. The pellet was then resuspended in 100 μl of the same buffer and stored at −80 °C until further use.

sEVs were measured for their protein content using a BCA protein assay kit (Pierce Protein Biology; Thermo Fisher Scientific Life Sciences). The presence of the sEVs was subsequently confirmed using a NanoSight NS300 (Malvern Instruments, Ltd., Malvern, U.K.) and transmission electron microscope (TEM). Detection of the sEV surface markers CD81 or CD63 was performed using western blotting.

### Extracellular vesicle cellular uptake analysis

Isolated sEVs were tested for their ability to enter cells. The sEVs were labeled with PKH67 using the Green Fluorescent Cell Linker Kit (Sigma–Aldrich, St. Louis, MO) according to the manufacturer’s protocol. The labeled sEVs were isolated using ultracentrifugation again to clear out the unbound PKH67 dye. PKH67-bound small EVs were incubated with OPLL or PLL cells for 6 h at 37 °C and then washed three times with PBS. The nuclei were counterstained with DAPI (10 μg/ml) for 20 min with Triton X-100 before the cells were observed under a fluorescence microscope (Olympus, Tokyo, Japan). The EV-free supernatant was also incubated with PKH67 and purified to serve as a non-EV control.

### Analysis of miRNA sequencing data and microRNA target prediction

For analysis of microRNA data of OPLL- and PLL-derived sEVs, we took advantage of miRNA high-throughput sequencing data obtained by NovelBio Bio-Pharm Technology Co., Ltd., which can be downloaded from the GEO dataset GSE113632. The microRNA sequencing data of OPLL and PLL cells, which were also obtained from NovelBio Bio-Pharm Technology Co., Ltd., were downloaded from the GEO dataset GSE69787 at https://www.ncbi.nlm.nih.gov/geo/. The raw counts of miRNA reads were further normalized by transcripts per million (TPM) values ((miRNA total reads/total clean reads) × 10^6^). For microRNA target prediction, we used the miRanda algorithm (http://www.mirbase.org), which evaluates the binding possibility based on the duplex binding energy. The TPM data of sEV miRNAs are listed in Supplementary Data 2.

### Dual-luciferase reporter assay

HEK293T cells (cat No.CL-0005, Procell Life Science&Technology Co.,Ltd, Wuhan, China) were seeded into 96-well plates for 24 h. A mixture of the pmiR‐RB‐REPORT dual‐luciferase vector (wild type or site mutated plasmid, GeneChem, Shanghai, China) and miRNA mimics or scramble control were cotransfected into cells, and a PRL-TK vector (carrying Renilla luciferase) served as an internal control (Promega, Madison, USA). Forty-eight hours after transfection, the Dual-Luciferase Reporter Assay System (Promega, Madison, USA) was used to detect luciferase activity. Light intensity was normalized to firefly luciferase.

### RNA extraction and real-time qPCR

Cell samples were washed twice with PBS and lysed in 750 μL of TRIzol (Invitrogen, Carlsbad, USA) per sample for total RNA extraction. For tissue samples, ~3 × 3 × 3-mm chunks of tissue were homogenized in 750 μL of TRIzol per sample. Total RNA was then extracted according to the manufacturer’s instructions and further reverse transcribed using a ReverTra Ace^®^ qPCR RT Kit (Toyobo, Osaka, Japan). Real-time PCR and analysis were performed as previously described^45^. Single-strand cDNA was analysed using SYBR Green master mix (Roche, USA) according to the manufacturer’s instructions, and the primer sequences used in this study are listed in Supplementary Data 3.

### MicroRNA transfection

To assess the role of miR-320e, chemically synthesized and modified miRNA mimics (agomir) of miR-320e or miR-10a-3p from GenePharma Corporation Co., Ltd. were transfected into the indicated cells using FuGENE^®^ HD transfection reagent (Promega, Carlsbad, CA, USA). Inhibitors of miR-320e or miR-10a-3p (antagomir) were also synthesized by GenePharma Corporation Co., Ltd., and transfected into various cells using FuGENE^®^ HD transfection reagent according to the manufacturer’s instructions.

### Evaluation of multipotency using tissue-specific staining

To determine the osteogenic properties of ligament cells, Alizarin red S staining (ScienCell, San Diego, USA) and alkaline phosphatase (ALP) activity assays (Sidansai, Shanghai, China) were assessed 3 weeks after osteogenic induction as previously described^6^. Briefly, cells were treated with osteogenic induction medium consisting of DMEM with 10% FBS, 25 mg/ml ascorbate-2 phosphate, 10^−8^ M dexamethasone, and 5 mM β-glycerophosphate (all from Gibco, USA) for 2 weeks. After induction, cells were fixed in 4% paraformaldehyde, washed with phosphate-buffered saline (PBS), and then stained according to the manufacturer’s instructions.

To evaluate the osteoclast formation ability of monocytes in response to different treatments, we used TRAP staining after osteoclast induction. After 20 days, cells were fixed in 4% paraformaldehyde and TRAP stained (Acid Phosphatase, Leukocyte Kit; Sigma–Aldrich, 387 A) according to the manufacturer’s instructions. TRAP-positive multinucleated cells containing 3 or more nuclei were identified as osteoclasts and counted under a light microscope (Olympus IC70).

For assessment of chondrogenic differentiation of MSCs in response to different treatments, we first fixed the cells in 4% paraformaldehyde after three weeks of induction. Then, the cells were stained with 2% Alcian blue (Sigma–Aldrich, USA) in 3% acetic acid solution (Sigma–Aldrich, USA) for 30 min to microscopically examine proteoglycans produced by the cells.

### Immunohistochemical analysis

Specimens were decalcified in 10% ethylenediaminetetraacetic acid (EDTA, pH 7.4) for 1 month, dehydrated and embedded in paraffin. Sections (5 μm) were cut and stained with haematoxylin and eosin (H&E) or TRAP by Wuhan Servicebio Technology Co., Ltd. For immunohistochemistry analysis, sections were blocked in 3% BSA for 30 min and then incubated with primary antibodies against TAK1 (ab109526, Abcam, USA) and OCN (23418-1-AP, ProteinTech, Wuhan, China) at a 1:100 dilution. Antigen retrieval was performed in 95 °C citrate buffer (pH 6) for 10 min, and then sections were incubated with primary antibody at 4 °C overnight. After processing with an ABC detection kit (Vector Laboratories, Burlingame, CA), sections were visualized under an Olympus BX51 light microscope equipped with an Olympus DP70 camera (Olympus Co., Tokyo, Japan) and quantified using ImageJ software version 1.53 (US National Institutes of Health).

### Immunofluorescence study

Small EVs were incubated with PLL cells for 6 h at 37 °C and then washed three times with PBS, then fixed with 4% paraformaldehyde. For the detection of CD81 and CD63, cell samples were blocked with 5% bovine serum albumin, then incubated with rabbit monoclonal anti-CD81 (1:100, ab219209, Abcam) or mouse monoclonal anti-CD63 (1:100, ab1318, Abcam) at 4 °C overnight. After stringency washes, cells were incubated with relative secondary antibodies (Goat Anti-Mouse IgG and Goat Anti-Rabbit IgG conjugated with Alexa Fluor 647, 1:1000, ab150115 and ab150079, Abcam) for 2 h. After washes, the nuclei were counterstained with DAPI (10 μg/ml) for 20 min before observing under a fluorescence microscope (Olympus, Tokyo, Japan).

### Western blots

Proteins were extracted using a commercial kit (No. C510003, Sangon Biotech, China) according to the manufacturer’s instructions. The following primary antibodies were used (all at a 1:1,000 dilution): rabbit anti-RUNX2 (ab23981, Abcam), rabbit anti-OSX (ab94744, Abcam), rabbit anti-OCN (23418-1-AP, ProteinTech, Wuhan, China), rabbit anti-ALP (ab83259, Abcam), rabbit anti-ACP5 (ab191406, Abcam), rabbit anti-ITBG3BP (ab192324, Abcam), rabbit anti-MMP9 (10375-2-AP, ProteinTech, Wuhan, China), rabbit anti-CTSK (ab19027, Abcam), rabbit anti-GAPDH (10494-1-AP, ProteinTech, Wuhan, China), rabbit anti-FOXO3 (10849-1-AP, ProteinTech, Wuhan, China) and rabbit anti-TAK1 (ab109526, Abcam). The protein samples were separated by 10% SDS–PAGE and subsequently transferred to nitrocellulose filter membranes (Pall Corp., Washington, NY) using a wet transfer blotting system (Bio-Rad, Hercules, CA). After incubation, a goat anti-rabbit-HRP secondary antibody (Pierce, USA) was applied at a 1:2,000 dilution. The proteins were then detected by a chemiluminescence detection system (Millipore, USA). Anti-GAPDH was used as an endogenous control. The uncropped scans of all blots were provided in the Source Data file.

### In vivo heterotopic bone formation assay and OPLL model analysis

For the in vivo heterotopic bone formation assay, we resuspended the posterior longitudinal ligament cells into coculture with Bio-Oss Collagen (Geistlich, GEWO GmbH, Baden, Germany) scaffold in osteogenic medium and treated them with equal amounts (10 µg) of OPLL-derived sEVs, PLL-derived sEVs or EFS for 2 days. Small EVs were purified using the Cell Culture Media Exosome Purification and RNA Isolation Maxi Kit (60900, Norgen Biotek Corp, Ontario, Canada) before treatment. Then, the seeded scaffolds were subcutaneously implanted on the backs of 4-week-old BALB/c homozygous nude (nu/nu, all male, Shanghai Model Organisms Center, Inc., China) mice (6 mice per group) randomly as previously described^6^. Six weeks later, the scaffolds were harvested and fixed in 10% neutral-buffered formalin for further analysis.

To assess OPLL development in vivo, we used tip-toe walking mice (TWY-ttw mice) for the experiment (Central Institute for Experimental Animals, CIEA, Kawasaki, Japan). Six-week-old ttw male mice were used in this study, and equal amounts (50 µg in a 100 µl volume was used for injection) of purified (60900, Norgen Biotek Corp, Ontario, Canada) OPLL-derived EVs, PLL-derived sEVs or EFS were injected into the tail vein of each mouse randomly. The injection was repeated at 2-day intervals for 1 week. All mice were sacrificed after 18 weeks unless they died during the experiment. The spines of all mice were harvested and subjected to micro-CT scanning and immunohistochemical analysis. During the experiment, all mice were observed each day for any gait imbalance or other ankylosing symptoms. The mice were kept in a controlled environment with 40% humidity, 20 °C temperature conditions and 12 h light-and-dark cycles, and were accessible at all times to water and food. All animal experiments were approved by the Navy Medical University Animal Care and Use Committee.

### Micro-CT analysis

The bioscaffolds or cervical spines of *ttw* mice were harvested after treatment and fixed in 10% neutral-buffered formalin. Micro-CT scanning and analysis were performed by Shanghai Model Organisms Centre Inc. (Siemens, Munich, Germany). Micro-CT images were processed by Inveon Research Workplace version 4.0 (Siemens Healthcare GmbH, Erlangen, Germany) to perform 3D reconstruction and volume quantification. The region of interest was selected within the bioscaffold to calculate bone volume (CT value above 2000 Hu)/total volume (CT value above 700 Hu) and bone mineral density (BMD, mg/ml). Sagittal spinal CT images with significant OPLL were used to calculate the occupation percentage of the spinal canal.

### Statistical analysis

Data are reported as the mean value ± SD (standard deviation). Data analysis was performed using SPSS version 16.0. Student’s *t* test and one-way ANOVA were performed where appropriate, and a *P* < 0.05 was considered statistically significant.

### Reporting summary

Further information on research design is available in the Nature Research Reporting Summary linked to this article.

## Supplementary information

Supplementary information

Reporting Summary

Description of Additional Supplementary Files

Supplementary Data 1

Supplementary Data 2

Supplementary Data 3

Supplementary Video 1

Supplementary Video 2

## Data Availability

The microRNA sequencing data of OPLL- and PLL-derived sEVs (Dataset GSE113632) and the microRNA sequencing data of OPLL and PLL cells (Dataset GSE69787) can be downloaded from GEO at https://www.ncbi.nlm.nih.gov/geo/ (Fig. 2C–I were generated using these sequencing data). Source data for Figs. 1–10 and Supplementary Figures 1–8 have been provided in the Source Data File to provide the necessary information needed to interpret, verify and extend the research in the article. All data and genetic material used for this paper are available from the authors upon request. Source data are provided with this paper.

## Source data

Source Data
