# Supplementary material for: Small extracellular vesicle-mediated miR-320e transmission promotes osteogenesis in OPLL by targeting TAK1
Source: Nat Commun. 2022 May 5;13:2467. doi: 10.1038/s41467-022-29029-6 (PMC9072352; doi:10.1038/s41467-022-29029-6)
Supplement: Supplementary file 1 — Supplementary information [file 41467_2022_29029_MOESM1_ESM.pdf]

SUPPLEMENTARY FILES

Small extracellular vesicle mediated miR-320e transmission promotes osteogenesis in OPLL by targeting TAK1

Chen Xu[1,*], Zicheng Zhang[1,2,3,*], Ning Liu[1,4,*], Li Li[3,*], Huajian Zhong[1], Ruizhe Wang[1], Qianghui Shi[1], Zifan Zhang[1], Leixin Wei[1], Bo Hu[1], Hao Zhang[5], Xiaolong Shen[1], Yue Wang[2], Yang Liu[1, §], Wen Yuan[1, §]

[1] Department of Orthopedics, Changzheng Hospital, Naval Medical University, Shanghai, 200433, China.

[2] Department of Orthopedics, the Fouth Medical Center of PLA General Hospital, Beijing, 100048, China.

[3] Department of Histology and Embryology, College of Basic Medicine, Naval Medical University, Shanghai, 200433, China.

[4] Department of Orthopedics, 923th Hospital of the Joint Logistics Support Force of PLA, Nanning, 530021, China.

[5] Department of Orthopedics, 967th Hospital of the Joint Logistics Support Force of PLA, Dalian, 116021, China.

§ Corresponding authors

* These authors contributed equally to this work

Contact Information of Corresponding Authors:

Prof. Yang Liu, Tel and Fax: +86-021-81870958, E-mail: yangliuchz@hotmail.com

Prof. Wen Yuan, Tel and Fax: +86-021-81870965, E-mail:yuanwenspine@smmu.edu.cn

SUPPLEMENTARY FILE CONTAINED THE FOLLOWING CONTENTS

Supplementary Data Files:

Supplementary Data 1 General information of patients for sample collection

Supplementary Data 2 Differentially expressed miRNAs In OPLL derived EVs

Supplementary Data 3 Oligonucleotide sequences used in this study

Supplementary Videos:

Supplementary Video 1 Typical lower limb symptom of ttw mice

Supplementary Video 2 Non-symptomatic gait of ttw mice

Supplementary Figures:

Supplementary Figures 1-8 in following pages.

Supplementary Figures & Legends

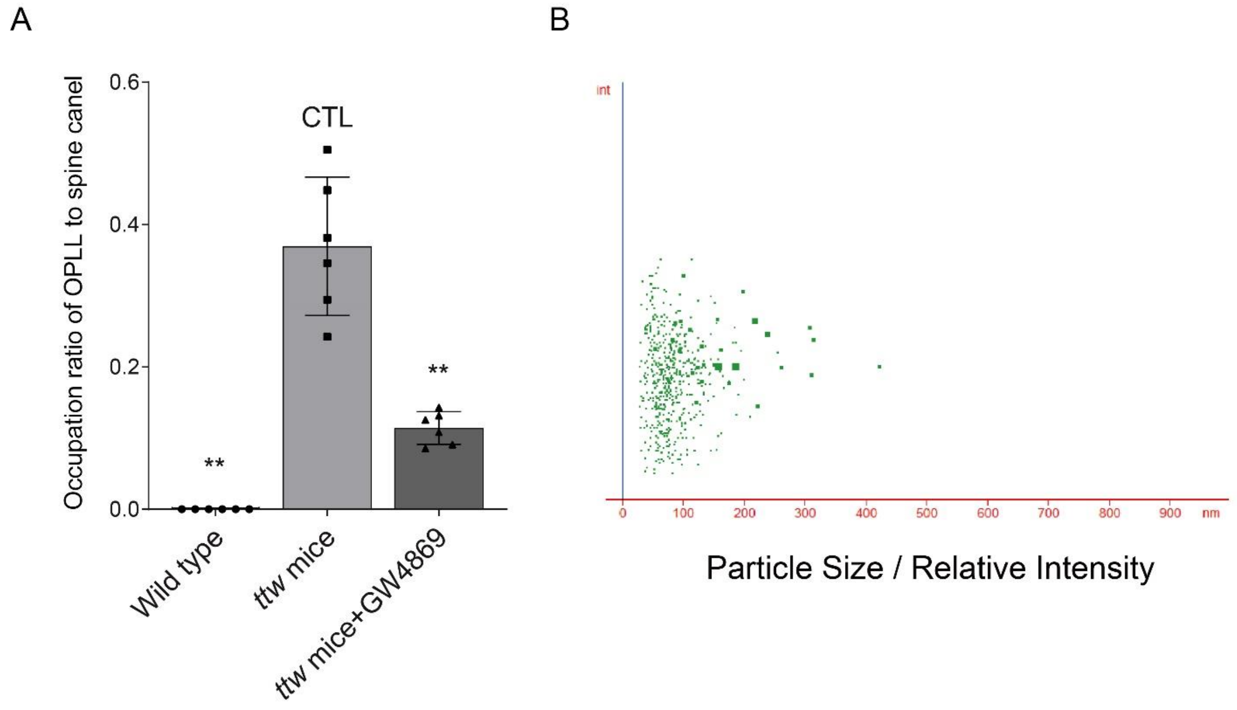

Supplementary Figure 1. Small EVs (sEVs) contribute to OPLL formation. (**A**) Quantification of the relative occupation ratio of the ossified posterior longitudinal ligament in the spinal canal of GW4869 (EV inhibitor) treated or none treated tip toe walking mice (*ttw*), the wild type ICR mice were used as non-OPLL controls. The area of the spinal canal is divided by the area of ossified posterior longitudinal ligament in the same cross section level of the spine to generate the relative occupational ratio of OPLL. Data were presented as mean ± SD, and all ratio data were compared to that in *ttw* mice group using two tailed t-test, n=6 mice for each group (18 male mice altogether), $^{**}p$ value < 0.001. (**B**) The Nanoparticle Tracking Analysis (NTA) result showing the particle size/relative intensity plot of the collected small extracellular vesicles. Note that most detected particles were around 100nm in size. Source data are provided as a Source Data file.

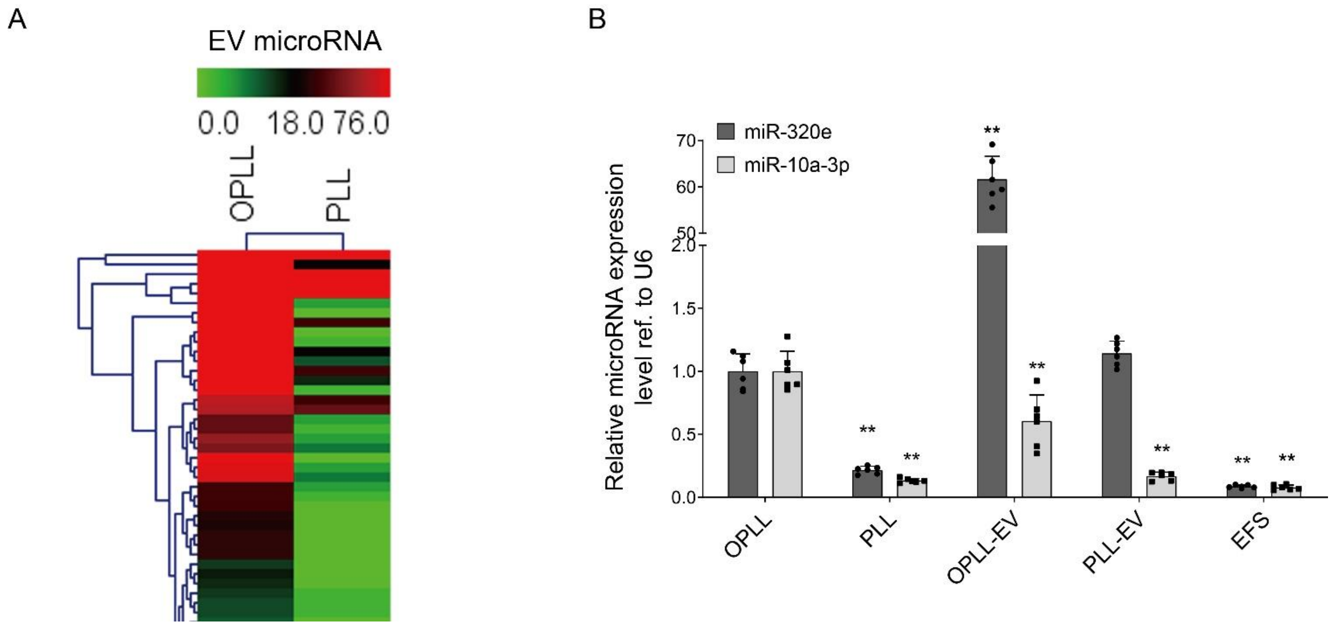

Supplementary Figure 2. miR-320e is an OPLL-sEV specific microRNA. (**A**) Hierarchical cluster showing the differentially expressed microRNAs in OPLL and PLL derived small EVs. (**B**) qRT-PCR analysis comparing the basal expression level of miR-320e and miR-10a-3p in different cell or sEVs under equal amount of total RNA extracted. Expression level of U6 was detected and served as internal reference. Data in each group was compared to that of OPLL cells (OPLL group) using two tailed t-test, and all data were presented as mean ± SD, n=6 biologically independent samples, $^{**}p < 0.01$. Detailed statistical data and source data are provided in a Source Data file.

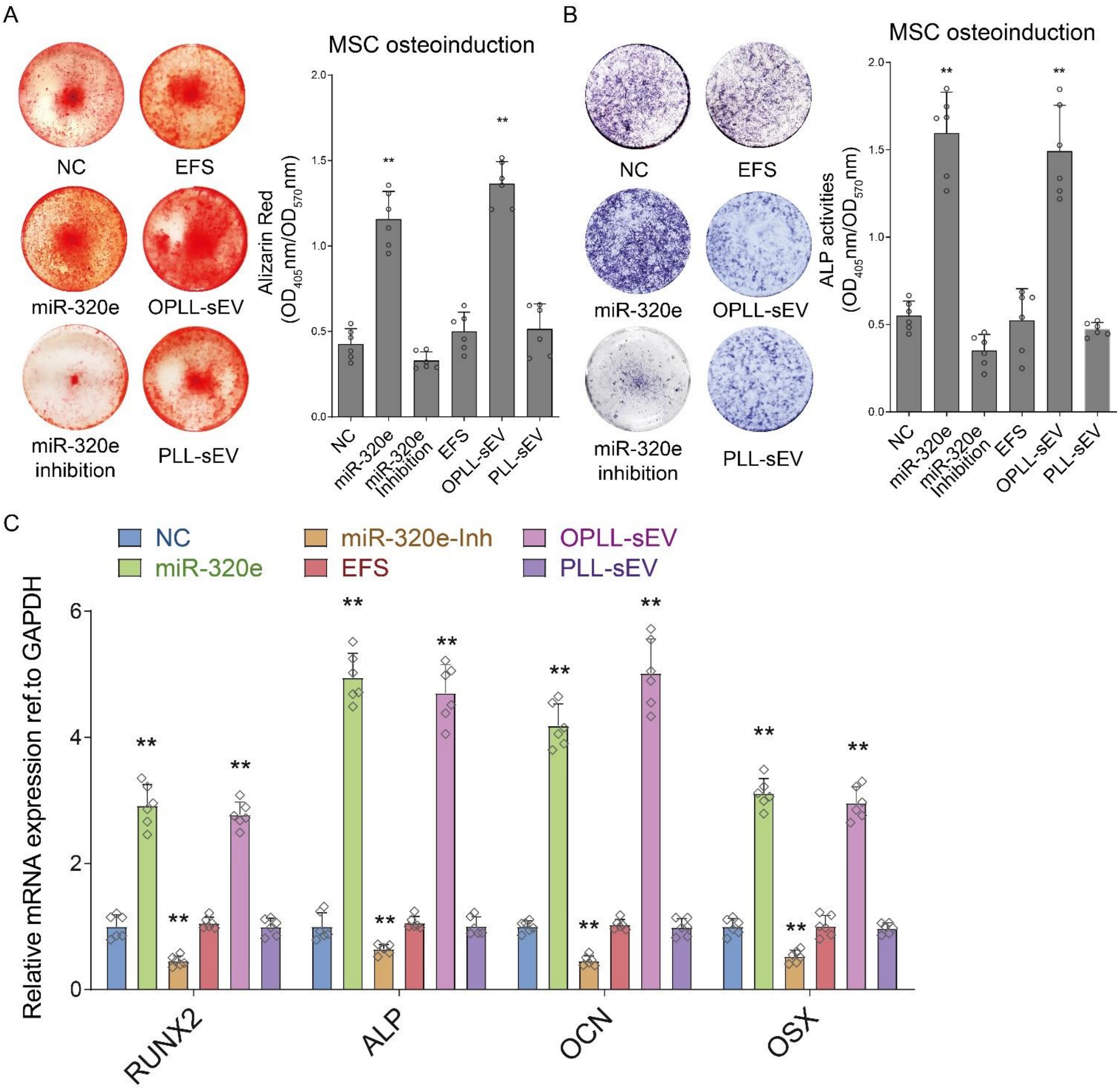

Supplementary Figure 3. OPLL-sEV and its specific miR-320e is functional in promoting MSC osteogenesis. The osteogenic properties of MSCs are analyzed using alizarin red staining (**A**), alkaline phosphatase staining (**B**) and qRT-PCR analyzing the osteogenic gene expressions (**C**, n=6, two-way ANOVA) after osteogenic induction for 21 days under varies kind of treatment. The colorimetric quantification is shown in the right panel in (**A**, n=6, one-way ANOVA) and (**B**, n=6, one-way ANOVA), respectively. NC group represents transfecting scramble control miRNA mimics. EFS group represents treating MSC with the supernatant of sEV collection that is free of sEVs. All data are compared to that of NC group, and shown as mean ± SD. $^{*}p$ <0.05, $^{**}p$ < 0.01. Detailed statistical data and source data are provided in a Source Data file.

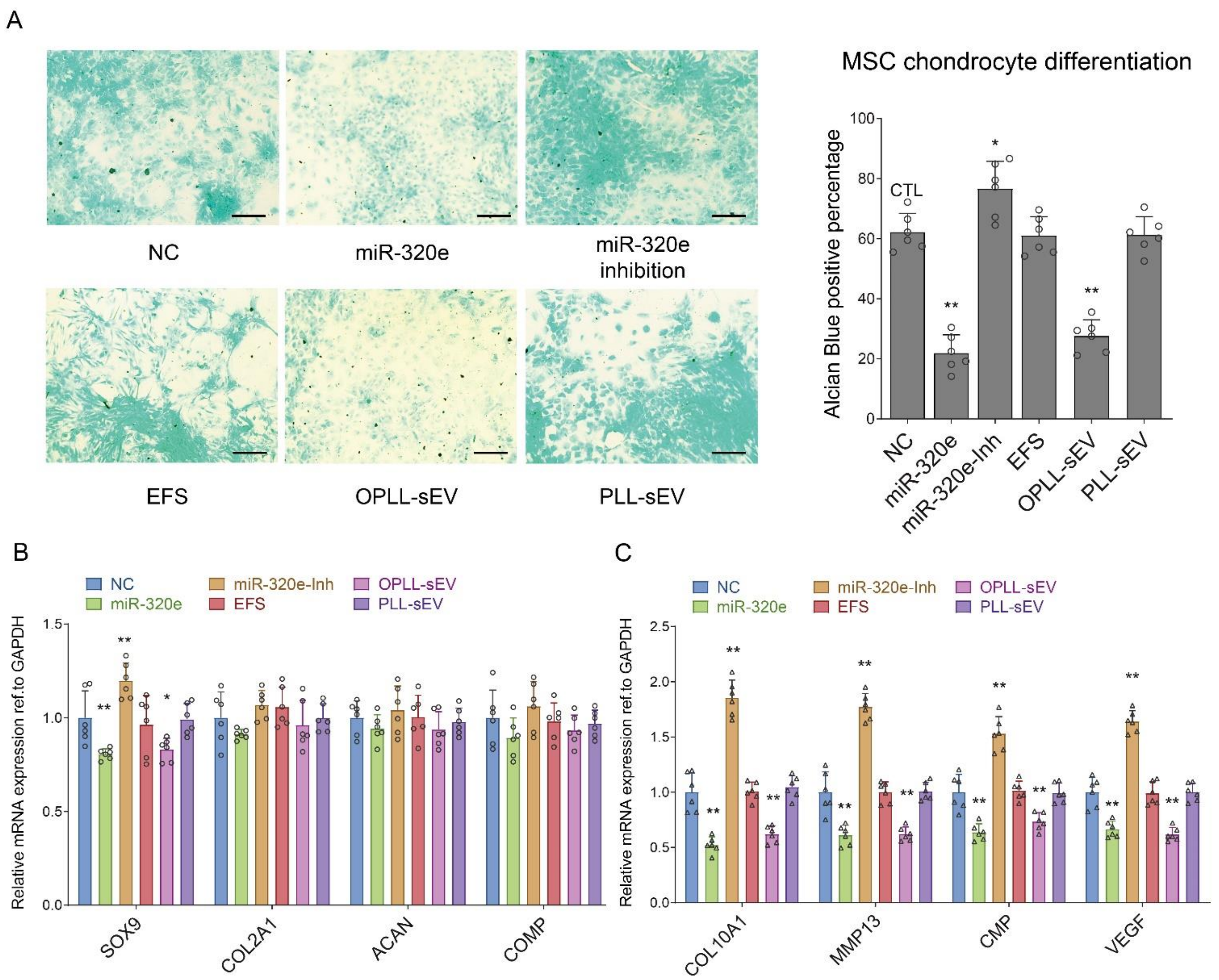

Supplementary Figure 4. OPLL-sEV and its specific miR-320e inhibits chondrogenesis in MSCs. The chondrogenic properties of MSCs are analyzed using Alcian blue staining (**A**, n=6) and qRT-PCR analyzing the early (**B**, n=6, two-way ANOVA) and late (**C**, n=6, two-way ANOVA) chondrogenic gene expressions after chondrogenic induction for 21 days under varies kind of treatment. Note that the early chondrogenic genes were not significantly affected except for SOX9, while the late chondrogenic genes were greatly affected by OPLL-sEV and miR-320e overexpression. The scale bars represent 200μm. The quantification of Alcian blue stained positive cells is shown in percentage at the right panel in (**A**, n=6, one-way ANOVA). NC group represents transfecting scramble control miRNA mimics. EFS group represents treating MSC with the supernatant of sEV collection that is free of sEVs. All data are compared to that of NC group, and shown as mean ± SD. $^{*}p < 0.05$, $^{**}p < 0.01$. Detailed statistical data and source data are provided in a Source Data file.

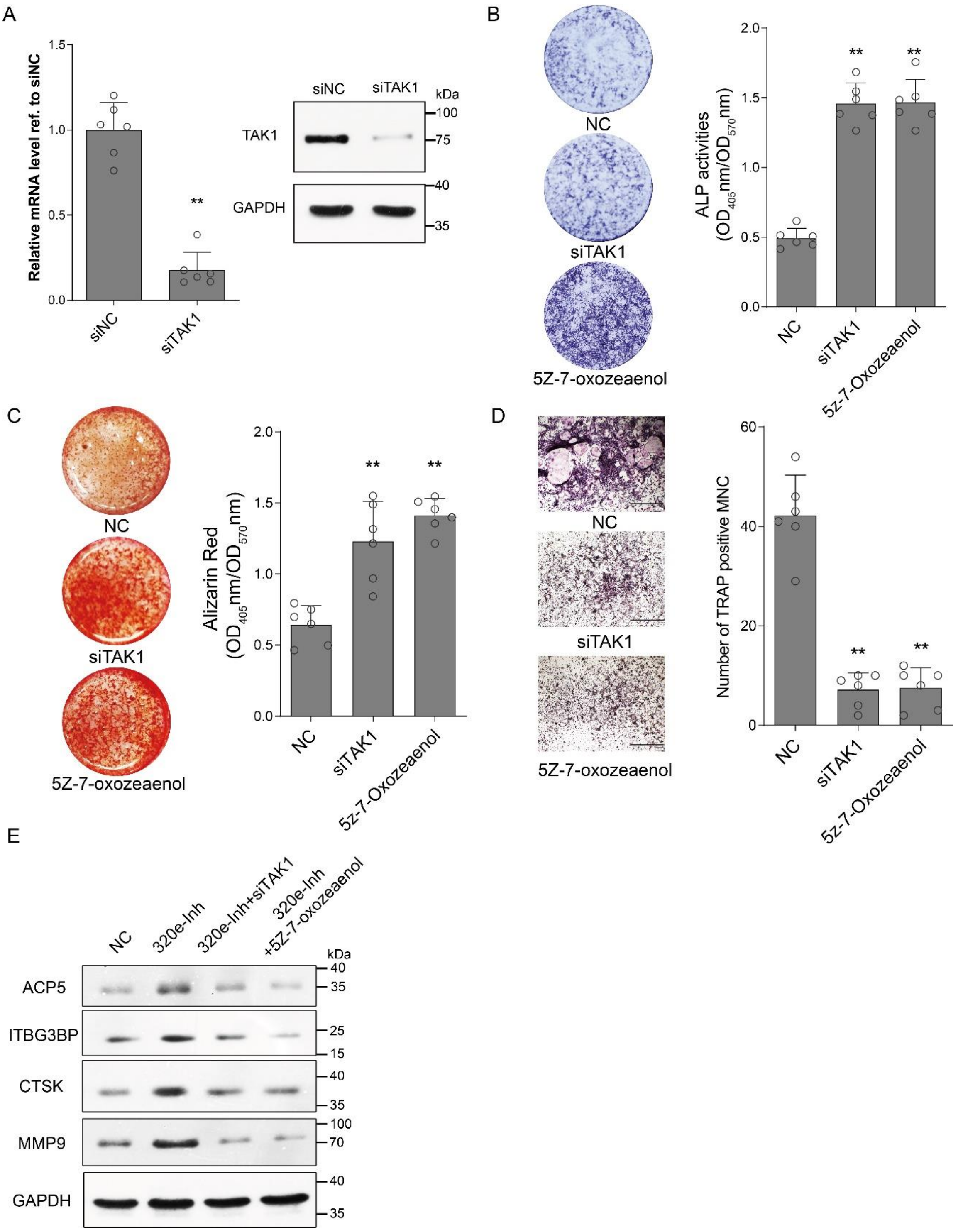

Supplementary Figure 5. TAK1 inhibits osteogenesis and promote osteoclast differentiation. (**A**) qRT-PCR analysis (n=6, t-test) and Western blot (n=3) confirming the knockdown efficiency of TAK1 targeting small interfering RNA in OPLL cells. The siTAK1 group is transfected with a combination of two TAK1 targeting siRNAs. The osteogenic properties of PLL cells are analyzed using alkaline phosphatase staining (**B**, n=6, one-way ANOVA) or alizarin red staining (**C**, n=6, one-way ANOVA) after osteogenic induction for 21 days in different groups. The colorimetric quantification is shown in the right panel respectively. 5Z-7-oxozeaenol (a TAK1 specific inhibitor) is used at 20nM to inhibit TAK1 activities. (**D**) Osteoclast differentiation was induced by treating the monocyte cells with 30 ng/ml M-CSF and 100 ng/ml RANKL. Cells were fixed and stained for Tartrate-resistant acid phosphatase (TRAP) activities at days 21. The quantification of osteoclast precursors is shown in the right panel, n=6, one-way ANOVA. The scale bars represent 500μm. (**E**) Western blot analysis confirmed the expression change of osteoclast differentiation related genes under different treatment in monocytes, n=3. All data are shown as mean ± SD. $^{*}p$ <0.05, $^{**}p$ < 0.01. Detailed statistical data and source data are provided in a Source Data file.

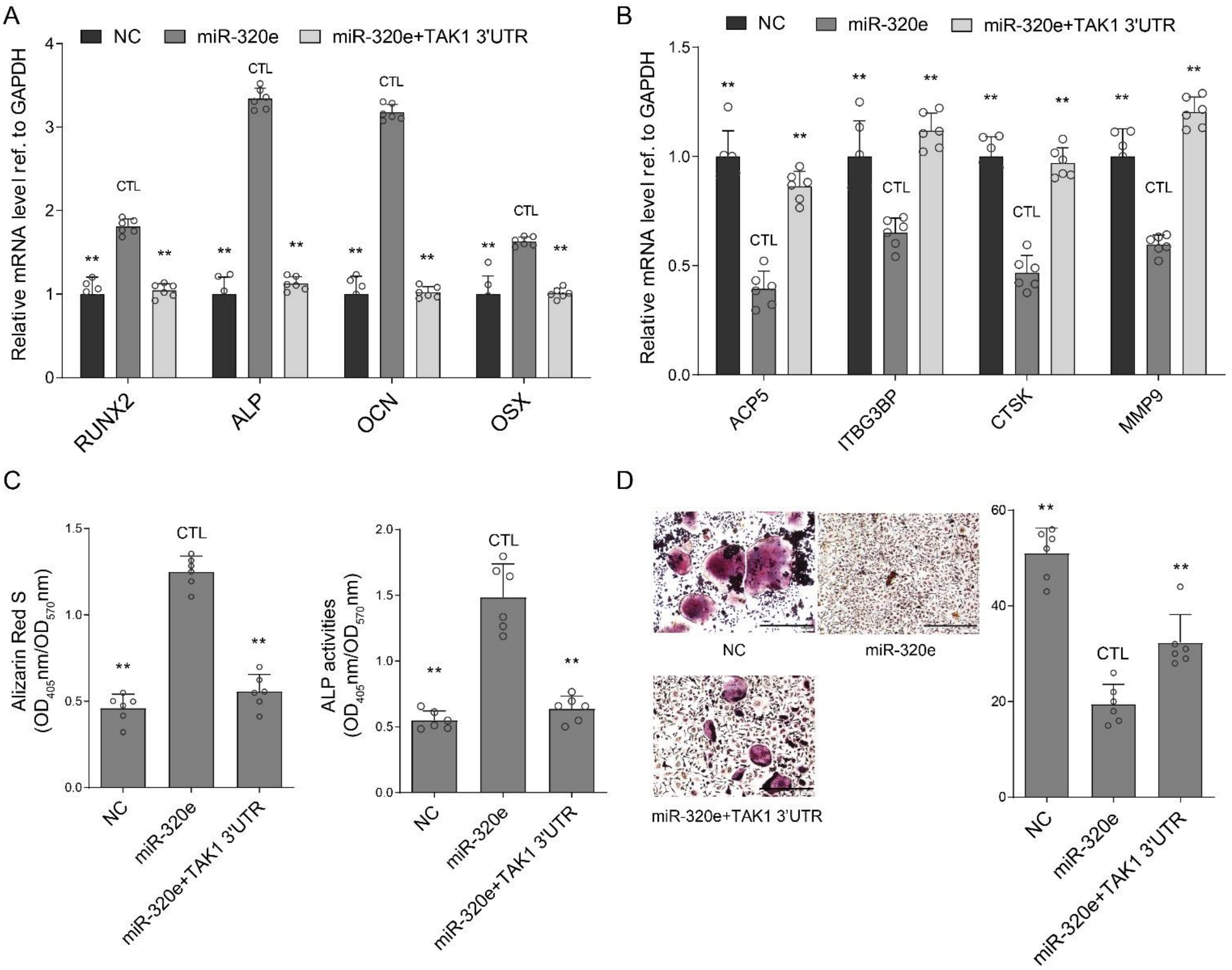

Supplementary Figure 6. TAK1 3'UTR effectively attenuated miR-320e's function in promoting osteogenesis and inhibit osteoclast differentiation. qRT-PCR analysis confirmed that TAK1 3'UTR overexpression by lentivirus vector could effectively reverse the osteogenic promoting effect (**A**, n=6, two-way ANOVA) and osteoclastogenic inhibiting effect (**B**, n=6, two-way ANOVA) of miR-320e overexpression in PLL cells (**A**) and monocyte (**B**) respectively. (**C**) This rescue effect of TAK1 3'UTR is further analyzed by assessing the osteogenic properties of PLL cells using alkaline phosphatase staining and alizarin red staining quantification after osteogenic induction for 21 days in different groups, both n=6, two-tailed test. (**D**) Osteoclast formation quantification using Tartrate-resistant acid phosphatase (TRAP) staining was also performed to confirm the rescue effect of TAK1 3'UTR on miR-320e overexpression in human monocyte, n=6, two-tailed test. The scale bars represent 500μm. All data are shown as mean ± SD. $^{*}p$ <0.05, $^{**}p$ < 0.01. Detailed statistical data and source data are provided in a Source Data file.

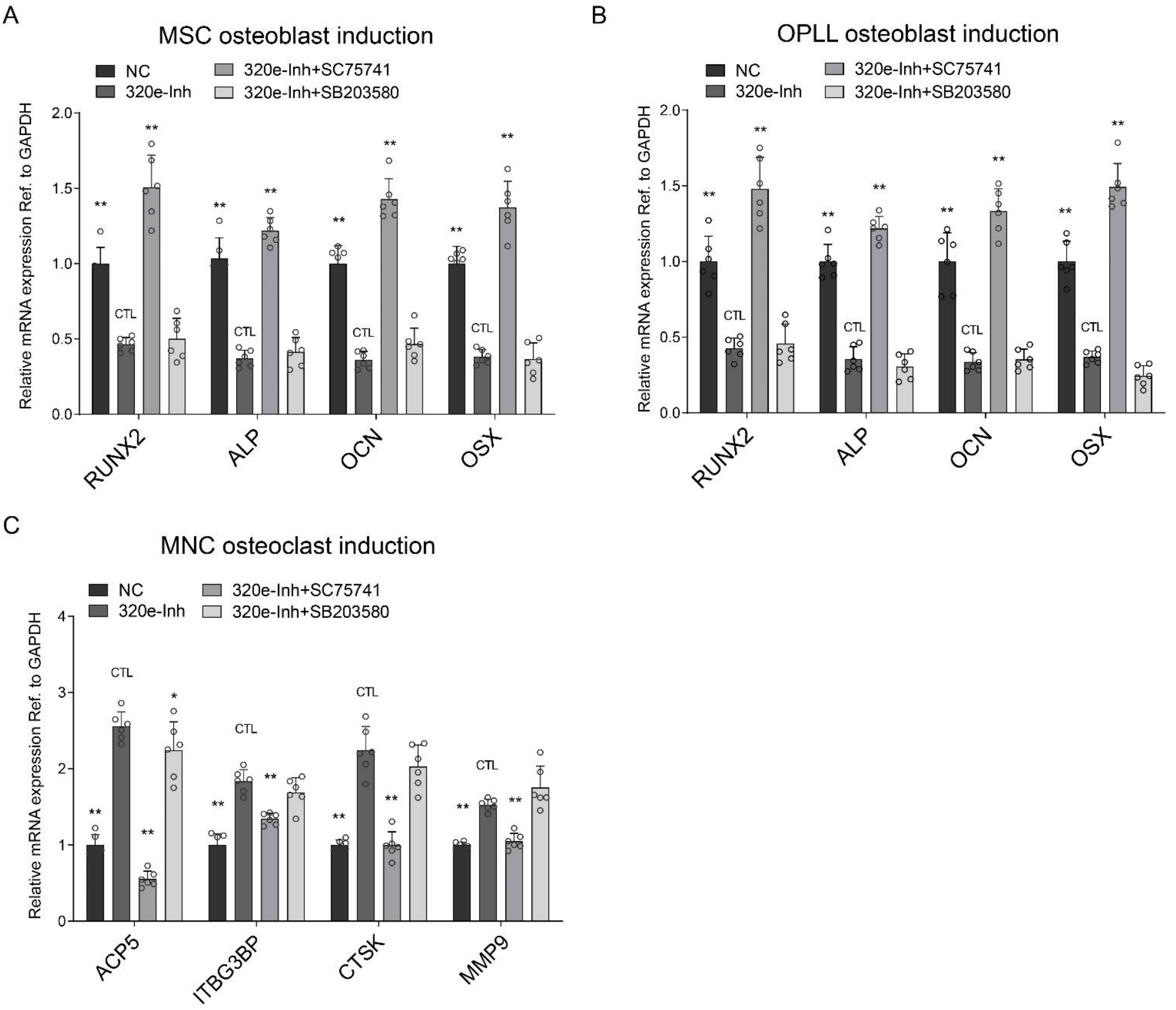

Supplementary Figure 7. NF-κB is required for miR-320e to exert osteogenic promoting function. qRT-PCR analysis showing the RNA levels of osteogenic related genes in osteogenic induced MSC (**A**, n=6, two-way ANOVA) and OPLL cells (**B**, n=6, two-way ANOVA) under different treatment. The SC75741 (200nM) is used to inhibit NF-κB pathway, and SB203580 (1nM) is used to inhibit the p38 MAPK pathway. 320e-Inh represents cells were transfected with miR-320e inhibitor. (**C**) qPCR analysis showing the RNA levels of osteoclastogenesis related genes in osteoclast induced monocytes (MNC), n=6, two-way ANOVA. Addition with SC75741 but not SB203580 significantly reversed the effect of miR-320e inhibition, which implied that NF-κB signaling is required for miR-320e's function. GAPDH level were detected and served as internal reference. All data are shown as mean ± SD. $^{*}p$ <0.05, $^{**}p$ < 0.01. Detailed statistical data and source data are provided in a Source Data file.

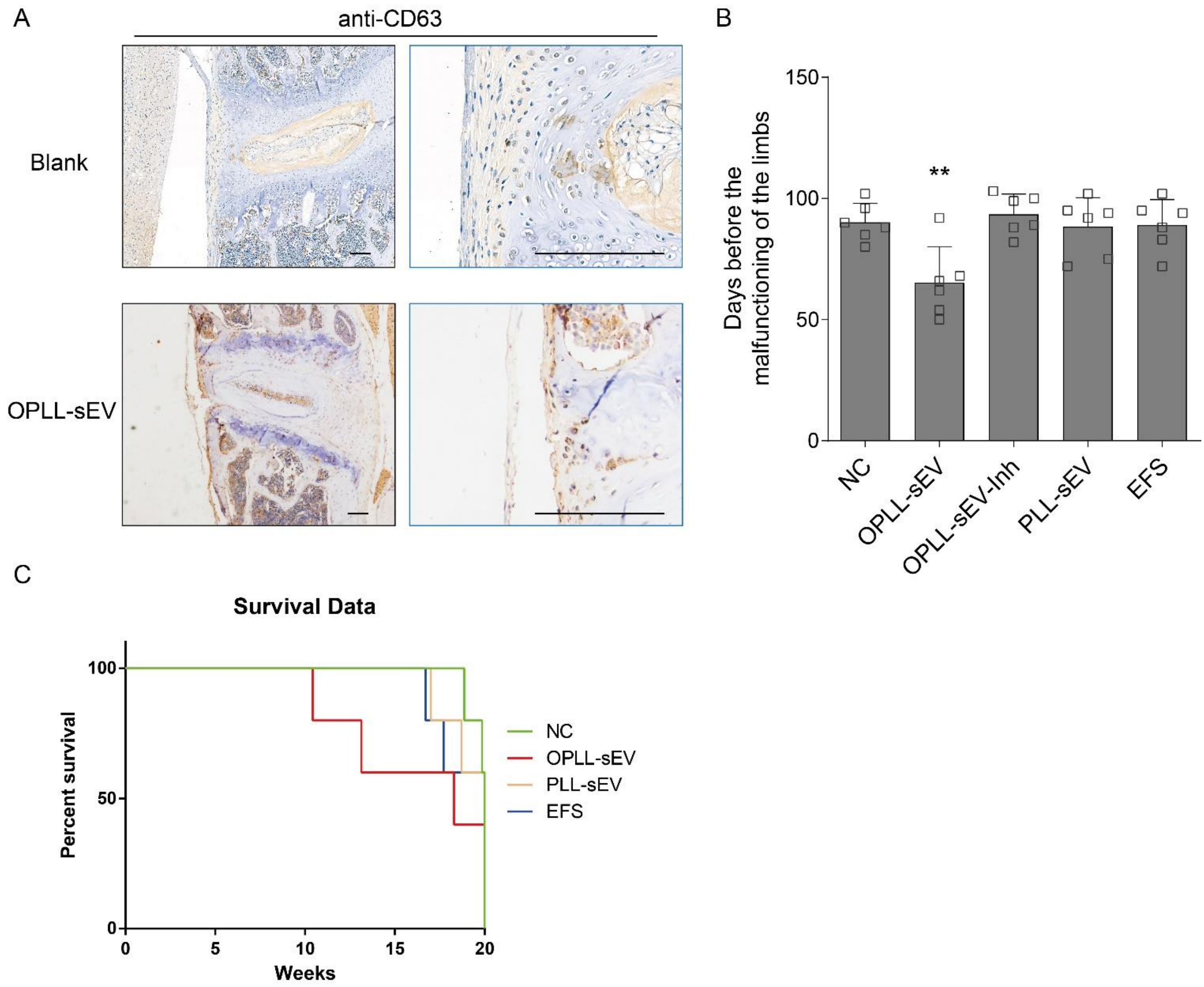

Supplementary figure 8. In vivo evidence showed OPLL-sEV could promote ossification and neurological symptom in *ttw* mice.

(**A**) immunohistochemistry analysis showing the distribution of humanized CD63 expression in OPLL-sEV injected spine tissue. Because OPLL-sEVs were derived from human ligament cells, mouse spine ligament cells that receive OPLL-sEV could be stained positive for anti-human CD63 antibodies. Note that in the Blank group, no cellular CD63 expression were found. While in OPLL-sEV injected group, the ligament region seemed to have clustered expression of human CD63, which presented that OPLL-sEV injected through mouse tail veins could efficiently arrive and affect the posterior longitudinal ligament. The scale bars represent 800μm. (**B**) Mean data of days that were free of neurological symptom since the first treatment in *ttw* mice between each group, n=6, one-way ANOVA. The function of lower limbs was observed every day to assess neurological defects in mice. (**C**) The survival data of *ttw* mice injected with OPLL derived sEVs or other treatment indicated, n=5 for each group. OPLL-sEV-Inh represents OPLL-sEVs transfected with miR-320e inhibitors, PLL-sEV represents the sEVs derived from PLL cells, while EFS represents the supernatant of sEV collection that is free of sEVs. Data are shown as mean ± SD. $^{*}p$ <0.05, $^{**}p$ < 0.01. Detailed statistical data and source data are provided in a Source Data file.
