# Supplementary material for: Small extracellular vesicle-mediated miR-320e transmission promotes osteogenesis in OPLL by targeting TAK1
Source: Nat Commun. 2022 May 5;13:2467. doi: 10.1038/s41467-022-29029-6 (PMC9072352; doi:10.1038/s41467-022-29029-6)
Supplement: Supplementary file 2 — Reporting Summary [file 41467_2022_29029_MOESM2_ESM.pdf]

nature research

Corresponding author(s): Wen yuan, Yang Liu

Last updated by author(s): Nov 7, 2021

Reporting Summary

Nature Research wishes to improve the reproducibility of the work that we publish. This form provides structure for consistency and transparency in reporting. For further information on Nature Research policies, see our Editorial Policies and the Editorial Policy Checklist.

Statistics

For all statistical analyses, confirm that the following items are present in the figure legend, table legend, main text, or Methods section.

n/a	Confirmed	
☐	☒	The exact sample size (n) for each experimental group/condition, given as a discrete number and unit of measurement
☐	☒	A statement on whether measurements were taken from distinct samples or whether the same sample was measured repeatedly
☐	☒	The statistical test(s) used AND whether they are one- or two-sided<br>*Only common tests should be described solely by name; describe more complex techniques in the Methods section.*
☐	☒	A description of all covariates tested
☐	☒	A description of any assumptions or corrections, such as tests of normality and adjustment for multiple comparisons
☐	☒	A full description of the statistical parameters including central tendency (e.g. means) or other basic estimates (e.g. regression coefficient) AND variation (e.g. standard deviation) or associated estimates of uncertainty (e.g. confidence intervals)
☐	☒	For null hypothesis testing, the test statistic (e.g. F, t, r) with confidence intervals, effect sizes, degrees of freedom and P value noted<br>*Give P values as exact values whenever suitable.*
☒	☐	For Bayesian analysis, information on the choice of priors and Markov chain Monte Carlo settings
☒	☐	For hierarchical and complex designs, identification of the appropriate level for tests and full reporting of outcomes
☒	☐	Estimates of effect sizes (e.g. Cohen's d, Pearson's r), indicating how they were calculated

Our web collection on statistics for biologists contains articles on many of the points above.

Software and code

Policy information about availability of computer code

Data collection	No software was used to collect the data
Data analysis	Data analysis was performed using SPSS version 16.0; histology sections were analyzed using ImageJ software version 1.53 (US National Institutes of Health); Micro-CT images were processed by Inveon Research Workplace version 4.0 (Siemens Healthcare GmbH, Erlangen, Germany); For microRNA target prediction, we used the miRanda algorithm (http://www.mirbase.org), which evaluates the binding possibility based on the duplex binding energy.

For manuscripts utilizing custom algorithms or software that are central to the research but not yet described in published literature, software must be made available to editors and reviewers. We strongly encourage code deposition in a community repository (e.g. GitHub). See the Nature Research guidelines for submitting code & software for further information.

Data

Policy information about availability of data

All manuscripts must include a data availability statement. This statement should provide the following information, where applicable:

- Accession codes, unique identifiers, or web links for publicly available datasets
- A list of figures that have associated raw data
- A description of any restrictions on data availability

The microRNA sequencing data of OPLL- and PLL-derived sEVs (Dataset GSE113632, https://www.ncbi.nlm.nih.gov/geo/query/acc.cgi?acc=GSE113632) and the microRNA sequencing data of OPLL and PLL cells (Dataset GSE69787, https://www.ncbi.nlm.nih.gov/geo/query/acc.cgi?acc=GSE69787) can be downloaded from GEO at https://www.ncbi.nlm.nih.gov/geo/ (Figures 2C-I were generated using these sequencing data). Source data are provided with this paper, the related data generated in this study are provided in the Source data file to provide the necessary information needed to interpret, verify and extend the research in the article.

Field-specific reporting

Please select the one below that is the best fit for your research. If you are not sure, read the appropriate sections before making your selection.

☒ Life sciences ☐ Behavioural & social sciences ☐ Ecological, evolutionary & environmental sciences

For a reference copy of the document with all sections, see nature.com/documents/nr-reporting-summary-flat.pdf

Life sciences study design

All studies must disclose on these points even when the disclosure is negative.

Sample size	no sample size calculation was performed. The ligament sample size were defined by concerning the parameters that needed to be analyzed, and since only expression of microRNA-320e is needed to be analyzed, we think that more than 10 samples in each group is sufficient. For cellular analysis, sample number or replicate number is defined according to the minimal biological repeats number needed for statistical analysis (n=3), and mostly we choose n=6 for our cellular experiment.
Data exclusions	No data were excluded from the analyses
Replication	For experiments done in this study, the replication is mostly done 6 times for each experiment (except for Western blot analysis, which 3 times of replication were done), and all replications were successfully done.
Randomization	In our study, cellular studies and human tissue data does not involve randomization, as cells were in uniform status and human tissues were collected from age matched patients. For animal studies, the mice were divided into each group randomly and followed by certain treatment. During which simple randomization method was used.
Blinding	The investigators were blinded during the experiments, and during which only numbers were used to define samples.

Reporting for specific materials, systems and methods

We require information from authors about some types of materials, experimental systems and methods used in many studies. Here, indicate whether each material, system or method listed is relevant to your study. If you are not sure if a list item applies to your research, read the appropriate section before selecting a response.

Materials & experimental systems

n/a	Involved in the study	
☐	☒	Antibodies
☐	☒	Eukaryotic cell lines
☒	☐	Palaeontology and archaeology
☐	☒	Animals and other organisms
☐	☒	Human research participants
☒	☐	Clinical data
☒	☐	Dual use research of concern

Methods

n/a	Involved in the study	
☒	☐	ChIP-seq
☒	☐	Flow cytometry
☒	☐	MRI-based neuroimaging

Antibodies

Antibodies used	Primary antibodies rabbit anti-RUNX2 (ab23981, Abcam), rabbit anti-OSX (ab94744, Abcam), rabbit anti-OCN (23418-1-AP, ProteinTech, Wuhan, China), rabbit anti-ALP (ab83259, Abcam), rabbit anti-ACP5 (ab191406, Abcam), rabbit anti-ITBG3BP (ab192324, Abcam), rabbit anti-MMP9 (10375-2-AP, ProteinTech, Wuhan, China), rabbit anti-CTSK (ab19027, Abcam), rabbit anti-GAPDH (10494-1-AP, ProteinTech, Wuhan, China), rabbit anti-FOXO3 (10849-1-AP, ProteinTech, Wuhan, China) and rabbit anti-TAK1 (ab109526, Abcam) were used (all at 1:1,000 dilution).<br>Secondary antibodies were also used in this study as following: goat anti-rabbit-HRP secondary antibody (31460, Pierce, USA), Goat Anti-Mouse IgG conjugated with Alexa Fluor 647 (ab150115, abcam) and Goat Anti-Rabbit IgG conjugated with Alexa Fluor 647 (ab150079, Abcam)
Validation	The primary antibodies anti-RUNX2 (ab23981, Abcam), rabbit anti-OSX (ab94744, Abcam), rabbit anti-OCN (23418-1-AP, ProteinTech, Wuhan, China), rabbit anti-ALP were validated in human and mouse subjects in this study and in previous studies (Sci Rep. 2018 Jun 15;8(1):9225; Theranostics. 2020 Jun 12;10(17):7492-7509).<br><br>Rabbit anti-ACP5 (ab191406, Abcam), rabbit anti-ITBG3BP (ab192324, Abcam), rabbit anti-MMP9 (10375-2-AP, ProteinTech, Wuhan, China), rabbit anti-CTSK (ab19027, Abcam), rabbit anti-GAPDH (10494-1-AP, ProteinTech, Wuhan, China), rabbit anti-FOXO3 (10849-1-AP, ProteinTech, Wuhan, China) and rabbit anti-TAK1 (ab109526, Abcam) were all validated in human and mouse subjects by other studies listed in the web (https://www.abcam.com/tartrate-resistant-acid-phosphatase-antibody-epr15556-ab191406.html; https://www.abcam.cn/itgb3bp-antibody-ab192324.html; https://www.ptglab.com/Products/MMP9-Antibody-10375-2-AP.htm; https://www.abcam.cn/Cathepsin-K-antibody-ab19027.html; https://www.ptglab.com/products/GAPDH-Antibody-10494-1-AP.htm;

https://www.ptglab.com/products/FOXO3A-Antibody-10849-1-AP.htm; https://www.abcam.cn/TAK1-antibody-EPR5984-ab109526.html)

During the study, we performed different dilution assay and negative control to validate the reactions of these primary antibodies to the cell or tissue prior to the final experiment.

Eukaryotic cell lines

Policy information about cell lines

Cell line source(s)	Human bone marrow mesenchymal stem cells (cat No.HUXMA-01001) from Cyagen Biosciences (Guangzhou, China); HEK-293T cells (cat No.CL-0005) from Procell Life Science&Technology Co.,Ltd.(Wuhan, China)
Authentication	For Human bone marrow mesenchymal stem cells , the cell line was authenticated by the manufacturer for the ability to differentiate into multiple cell lineages and tested the surface markers of CD29, CD44, CD73, CD105 to be positive, and CD34, CD45, CD11 to be negative by flow cytometry, which comply with mesenchymal stem cells characteristics. For HEK-293T cells, the cell line was authenticated by the manufacturer (ATCC) and identified using human STR cell markers by sequencing
Mycoplasma contamination	the manufacturer and the authors all tested that no mycoplasma contamination was found in the cell line.
Commonly misidentified lines (See ICLAC register)	No misidentified lines were used in this study.

Animals and other organisms

Policy information about studies involving animals; ARRIVE guidelines recommended for reporting animal research

Laboratory animals	30 4-week-old ICR and 30 6-week old ttw mice (all male mice, Central Institute for Experimental Animals, CIEA, Kawasaki, Japan) were used. 30 4-week-old BALB/c homozygous nude (all male, Shanghai Model Organisms Center, Inc., China) were also used in this study. The mice were kept in a controlled environment with 40% humidity, 20°C temperature conditions and 12h light-and-dark cycles, and were accessible at all times to water and food.
Wild animals	No wild animals were used in this study
Field-collected samples	No field collected samples were used in this study
Ethics oversight	All the experimental protocols were approved by the Ethics Committee of Naval Medical University (Approval number:2019SL044). And all animal experiments were performed under the ARRIVE guidelines.

Note that full information on the approval of the study protocol must also be provided in the manuscript.

Human research participants

Policy information about studies involving human research participants

Population characteristics	16 OPLL patient tissue samples (11 males and 5 females, aged 47–65 years, mean age 53.8 years) and 12 PLL patient samples (7 males and 5 females, aged 48–64 years, mean age 54.2 years) were collected during anterior cervical corpectomy. All patients received anterior cervical corpectomy surgeries for cervical spondylotic myelopathy. No family history of genetic diseases were observed in these patients.
Recruitment	Patients with OPLL or patients that is going to receive anterior cervical corpectomy surgeries for cervical spondylotic myelopathy were asked to participate in this study to collect their ligament tissue during surgery. The age of patients were restricted to 45-65 years, and patients with other genetic disease like DISH was excluded in this study. The recruitment was done by physicians that does not participate in this study, and was blinded to the study content. No compensation was given or received for the participants and researchers.
Ethics oversight	All the experimental protocols were approved by the Ethics Committee of Naval Medical University (Approval number:2019SL044).

Note that full information on the approval of the study protocol must also be provided in the manuscript.
