# Supplementary material for: Small extracellular vesicle-mediated miR-320e transmission promotes osteogenesis in OPLL by targeting TAK1
Source: Nat Commun. 2022 May 5;13:2467. doi: 10.1038/s41467-022-29029-6 (PMC9072352; doi:10.1038/s41467-022-29029-6)
Supplement: Supplementary file 3 — Description of Additional Supplementary Files [file 41467_2022_29029_MOESM3_ESM.pdf]

Title: Supplementary Data 1
Description: General information of patients for sample collection

Title: Supplementary Data 2
Description: Differentially expressed miRNAs In OPLL derived EVs

Title: Supplementary Data 3
Description: Oligonucleotide sequences used in this study

Title: Supplementary Video 1
Description: Typical lower limb symptom of ttw mice

Title: Supplementary Video 2
Description: Non-symptomatic gait of ttw mice
